# A composite network of conserved and tissue specific gene interactions reveals possible genetic interactions in glioma

**DOI:** 10.1371/journal.pcbi.1005739

**Published:** 2017-09-28

**Authors:** André Voigt, Katja Nowick, Eivind Almaas

**Affiliations:** 1 Network Systems Biology Group, Department of Biotechnology, NTNU - Norwegian University of Science and Technology, Trondheim, Norway; 2 Bioinformatics Group, Department of Computer Science, and Interdisciplinary Center for Bioinformatics, University of Leipzig, Leipzig, Germany; 3 Bioinformatics, Institute of Animal Science, University of Hohenheim, Stuttgart, Germany; 4 Human Biology, Institute for Biology, Free University Berlin, Berlin, Germany; 5 K.G. Jebsen Center for Genetic Epidemiology, Department of Public Health and General Practice, NTNU - Norwegian University of Science and Technology, Trondheim, Norway; University of California San Diego, UNITED STATES

## Abstract

Differential co-expression network analyses have recently become an important step in the investigation of cellular differentiation and dysfunctional gene-regulation in cell and tissue disease-states. The resulting networks have been analyzed to identify and understand pathways associated with disorders, or to infer molecular interactions. However, existing methods for differential co-expression network analysis are unable to distinguish between various forms of differential co-expression. To close this gap, here we define the three different kinds (conserved, specific, and differentiated) of differential co-expression and present a systematic framework, CSD, for differential co-expression network analysis that incorporates these interactions on an equal footing. In addition, our method includes a subsampling strategy to estimate the variance of co-expressions. Our framework is applicable to a wide variety of cases, such as the study of differential co-expression networks between healthy and disease states, before and after treatments, or between species. Applying the CSD approach to a published gene-expression data set of cerebral cortex and basal ganglia samples from healthy individuals, we find that the resulting CSD network is enriched in genes associated with cognitive function, signaling pathways involving compounds with well-known roles in the central nervous system, as well as certain neurological diseases. From the CSD analysis, we identify a set of prominent hubs of differential co-expression, whose neighborhood contains a substantial number of genes associated with glioblastoma. The resulting gene-sets identified by our CSD analysis also contain many genes that so far have not been recognized as having a role in glioblastoma, but are good candidates for further studies. CSD may thus aid in hypothesis-generation for functional disease-associations.

## Author summary

With the ever increasing availability of large sets of gene expression data, much effort has been directed towards studying shared expression patterns between different genes. We have developed a general method for studying the variation of gene co-expression between two different conditions, which allows for a more detailed description and classification of interactions than previous methods. Applying our method to compare data from two different parts of the brain (the cortex and the basal ganglia), we find that it identifies genes known to be involved in key brain functions. Our analysis also identifies connections between a variety of genes previously known to be involved in the progression of glioma. Our method can also be applied in studies comparing between healthy and disease states, treatment and controls, among others.

## Introduction

How can genomic information that is the same in each cell of an individual be translated into a variety of cell and tissue types? It is clear that gene-regulatory mechanisms must play a leading role in differentiation processes. Transcription factors (TF) belong to the class of proteins that are able to regulate the expression of other genes. However, it is the combinatorial interactions of TFs at the promoter of a gene that determine if that gene is activated, repressed, or not regulated at all [1]. For instance during development, a tightly coordinated cascade of TFs is responsible for the activation and repression of genes that determine cell fate. The same is true for tissue specificity.

The ever-increasing availability of genome-scale microarray and sequencing data has led to the development of an array of methods to investigate cells and tissues at the systems level. One class of such methods, gene co-expression analyses, has found wide use by combining microarray studies with network theory [2–9]. Investigations using a variety of network methods have found that co-expression patterns often are correlated with biologically relevant processes, such as protein-protein interactions, regulatory cascades, and biological pathways [10–16]. Because of the frequently observed relationship between co-expression and function, co-expression analyses have been used in a variety of applications. Examples include functional annotation of genes [17], identification of pathways associated with diseases, such as Alzheimer’s [18] and autism spectrum disorder [19], as well as inference of molecular interactions [20]. It should be noted, however, that co-expressed gene pairs do not necessarily reflect direct biological interactions: Even direct transcriptional relationships may simply be the result of accidentally matched DNA motifs without any particular function [5]. In order to facilitate the study of gene co-expression, a variety of computational tools, notably WGCNA ([21]), have been made publicly available for general use.

A more recent development is the study of differential co-expression networks, which seeks to identify condition-specific co-expression patterns often associated with dysfunctional regulation [22, 23]. Many methods have been developed to generate such networks based on the implementation of different principles. In broad terms, the differential co-expression network methods can be divided into two groups. In the first group, the approaches typically generate co-expression networks that are specific to each condition studied [24–26]. Here, genes are connected by links if their co-expression score fulfills a set of statistical criteria for significance. It is a fairly straightforward matter to compare the resulting networks and subsequently, to extract interactions that are present in only one of the conditions or to identify genes subject to extensive rewiring.

The second group of methods is instead focused on assigning a score for each possible gene pair, after which the score is used as input in a process to determine whether there is a significant change in co-expression between the (possibly multiple) conditions. These scores may be as mathematically simple as the difference between a gene-pair’s Pearson or Spearman correlation over the conditions [27], or it may include additional steps to normalize the data [28–30]. Some of these methods determine group-wise co-expression by use of e.g. hierarchical clustering on correlation matrices [31] or decomposition of dependencies into global and group-specific components [32].

As the term “differential” suggests, the aim of both groups of methods is to identify *differences* in collective co-expression patterns in order to elucidate processes specifically relevant to a given condition. One example application of differential co-expression analysis is to identify target genes for treatment of a particular disorder (e.g. a specific type of cancer) by identifying genetic interactions potentially linked to harmful outcomes [29]. These interactions, or some of the interacting genes, may be disabled through appropriate means. However, should any of those genes also be involved in processes that are important under normal conditions, the suggested approach might run the risk of incidentally harming healthy cells and tissues. Consequently, the identification of genes that are potential disease-targets should be refined in such a way that it is possible to determine both conserved and differential co-expression in order to get a more comprehensive understanding of involved mechanisms.

While the various methods differ in the measures by which they identify differential co-expression, there is also considerable variety in what sort of data they produce. Some methods only seek to identify prominent differentially co-expressed genes, without considering the genes with which they are connected [26, 33]. Of the methods that seek to identify inter-gene relationships, some primarily focus on identifying communities or modules of genes that are collectively closely connected [28, 30, 34], while others provide a more general network of differentially co-expressed genes. The latter methods can be divided into unweighted networks [25] (also known as hard thresholding), in which all links are considered equal as long as they fulfill given criteria, or weighted [33] (also known as soft thresholding), where links are given a numerical score quantifying their prominence. These edge weights typically represent the magnitude of change in correlation between conditions. Unweighted networks can readily be converted into weighted networks by determining a specific cut-off value for the edge weight, and uniformly setting the weight of all edges above this cut-off to 1, while the remaining edge weights are set to 0. It has been proposed that gene co-expression networks should follow scale-free topologies [35], but this requirement is not universally imposed.

Just as the various approaches to differential gene co-expression differ in how co-expression is determined, there are fundamentally distinct types of differential co-expression to consider. While some methods characterize differential co-expression by correlations exclusive to a given condition and others by net changes in pairwise correlations, it is important to acknowledge that these two scenarios are not entirely interchangeable. To illustrate this point, we consider the following example: Assume that a pair of genes exhibit positively correlated expression under condition *α* and uncorrelated expression under condition *β*. In this case, both classes of approaches should, in principle, be able to identify a differential co-expression.

If we instead consider a pair of genes whose expression is positively correlated under condition *α* and negatively correlated under condition *β*, the two classes of methods will return different results: Methods based on net change in pairwise correlations should readily identify this gene pair as differentially co-expressed, and find the pair to be even more strongly linked than in the first example since the net change now may be larger. In contrast, methods based on determining differential co-expression by comparing unsigned co-expression networks from individual conditions [25, 26] would not find the pair of genes to be differentially co-expressed, provided that the absolute values of the correlations are not too dissimilar. However, current “net change” methods will not be able to qualitatively distinguish between the case of positive correlation under condition *α* and no correlation under condition *β*, and the case of positive correlation under condition *α* and negative correlation under condition *β*, even though they are fundamentally different in the type of genetic correlation. In the first case, differential co-expression might suggest concerted action between the genes under condition *α* and independent operation under condition *β*. In the second case, differential co-expression suggests interactions under both conditions, but with possibly different mechanisms in play.

Here, we will make a distinction between the two forms of differential co-expression in order to clearly distinguish between these two qualitatively different cases: Specific co-expression, which we will denote S, in which a gene pair is correlated under only one condition. This corresponds to the first example. Differentiated co-expression, denoted D, in which a gene pair is correlated in both tissues, but with opposing signs: In one condition, the correlation is positive and in the other condition it is negative. This corresponds to the second example. We propose a differential co-expression framework that allows the simultaneous determination and quantification of both conserved co-expressions, C, and the S- and D-types of differential co-expression patterns. Thus, a gene pair that is significantly co-expressed in one tissue may either be similarly co-expressed (C), co-expressed but with an opposite sign (D), or not show any significant co-expression when studied in another tissue (S).

In order to provide a more complete framework for differential co-expression analysis, we have developed an approach, called CSD (“Conserved, Specific, Differentiated”), to categorize gene pairs according to mathematically defined scores which will allow us to construct a unified differential co-expression network from experimental data. A systematic comparison between this new method and pre-existing methods is presented under Materials and Methods. We apply the method using two types of tissues: cerebral cortex and basal ganglia from a published data set containing samples from a large number of human individuals [36]. From this network, we identify potential key sets of interactions and groups of genes which may help explain functional differences across these two tissues.

## Materials and methods

### Gene expression data set

We have used the differential co-expression profiles in the GTEx V4 data set [36], focusing on tissue groups that (1) exhibit a high degree of similarity (as established by Pierson *et al.* [37]), and (2) for which the GTEx data set contains a sufficiently large number of samples.

Our data set 1 named *cortex*, consists of the GTEx groups: Brain—Cortex, Brain—Anterior cingulate cortex (BA24), and Brain—Frontal cortex (BA9). Our data set 2 named *basal ganglia* consists of the GTEx tissue types: Brain—Caudate (basal ganglia), Brain—Nucleus accumbens (basal ganglia), and Brain—Putamen (basal ganglia). These groupings add up to 73 data points for cortex, and 92 data points for the basal ganglia.

The GTEx V4 data set contains expression data for a total 55,993 loci. We have restricted our analysis to protein-coding open reading frames (as annotated in Ensembl), leaving a set of 18,453 genes (see S4 Text).

### Estimated variance in gene co-expression

We calculate the pairwise gene co-expression scores *ρ*_*ij*,*k*_ as Spearman rank-correlations for the pair of genes *i* and *j* in tissue *k* over all the gene expression data points *N*. As our analysis bases itself on identifying changes in *ρ*_*ij*,*k*_ between conditions, we need to determine the extent of the variability of *ρ*_*ij*,*k*_ within a condition due to confounding factors. While methods exist to evaluate the variance of computed Spearman correlations, we need to account for the fact that specific confounding factors may change the “actual” correlation within specific subpopulations of samples in a given condition.

To illustrate the problem, consider a hypothetical gene-pair shown to exhibit moderate co-expression in a specific type of tissue across a whole population. Upon detailed review of the data, it turns out that this gene-pair displays very high co-expression for some particular subgroups of the population (for instance, certain age groups, or in individuals suffering from certain diseases). At the same time, this pair is not showing a strong co-expression outside of these subgroups. In this hypothetical case, it would be difficult to tell if an observed difference in co-expression in another tissue and another population relates to differences between the tissues, or is due to confounding factors.

On the other hand, if the genes are consistently (but not particularly strongly) co-expressed across all possible groupings of individuals, we can say with greater confidence that the correlation reflects genuine control related to the condition (in this case, a tissue type). Consequently, we may attribute any difference of co-expression (if similarly consistent) in another population and another condition to the differences between the conditions.

In order to determine the variance in co-expression within each given tissue, we compute the Spearman rank-correlation $r_{ij,k}^{l}$ for each independent sub-sample *l* of size *n* drawn from the *N* data points. We use the standard error of the mean, *σ*_*ij*,*k*_ calculated from the set of $r_{ij,k}^{l}$ values, as a measure of intra-tissue co-expression variation. In order to achieve as many sub-samples as possible, increasing the chance of matching with particular confounding conditions, and accurately determine *σ*_*ij*,*k*_ while ensuring independence between the different sub-samples, we implement the following approach for selecting sub-samples:

1. The *N* data points (per gene) for the full sample are ordered and sequentially numbered.
2. The *N* data points are divided into sub-samples of size *n*. For instance, if *N* = 100 data points with a chosen sub-sample size *n* = 8, we initially create 12 sub-samples of size *n*, consisting of the data points 1-8, 9-16, 17-24 etc.
3. Beginning with data point *N* = 1 as initiating data point n*, we sequentially iterate through the data points, adding to the current sub-sample any data point that has not previously co-occurred in a sub-sample with any of the points already in the current sub-sample.
4. When the size of the current sub-sample reaches *n*, we re-initiate a new sub-sample with initiating data point n* and repeat step 3.
5. When no valid sub-sample of size *n* can be drawn with n* as the initiating data point, we choose n* = n* + 1 as the next initiating data point and repeat from step 3.
6. The approach is completed when n* = *N* and no more allowed sub-samples of size *n* can be constructed.

An example of the application of this algorithm is presented in S1 Text. When implementing this algorithm, we are ensured that two data points only co-occur once in a sub-sample. Note that, it is quite beneficial to select a sub-sample size *n* such that *n*^2^ = *N*, as this greatly increases the amount of possible sub-samples that will be generated. On the other hand, a small sub-sample size *n* makes for a coarse Spearman correlations, i.e. to achieve three-digit accuracy, a sub-sample size of 7 is recommended. Consequently, *N* = 49 data points is a recommended minimum in order to determine the standard error of the correlation *ρ*_*ij*,*k*_ within a given tissue (or more generally, a given condition) within reasonable accuracy. We recognize that for certain conditions (such as those involving rare diseases or large animals which may be difficult to acquire and maintain) this requirement might not be realistically fulfilled. In this case, subsampling may be omitted, and *σ*_*ij*,*k*_ set to 0 for that condition (or 1, if subsampling isn’t possible for either condition).

Additionally, while sub-sampling may be omitted in the event of few available data points, there would still need to be enough data points available to accurately determine the base correlation for the set. In order to determine a reasonable minimum, one should keep in mind that for two factually uncorrelated random sequences of *N* data points, there is still a likelihood 1/*N*! that the computed Spearman correlation is 1. As real-life gene expression data frequently involve thousands of genes, and thus millions of gene pairs, a small *N* can thus lead to a substantial number of false positives, no matter how stringent the required Spearman correlation. For instance, if we have a data set containing 1000 genes, and 8 expression values for each, we would expect at least 10^3^(10^3^ − 1)/(2 ⋅ 8!) ≈ 12 perfectly correlated gene pairs by pure chance in addition to any factually correlated gene pairs. While the confidence with which any perfect (or near perfect) correlation can be said to be biologically relevant depends on the total number of observed correlations, users should be aware of these caveats when trying to draw conclusions based on small sample sizes. Additionally, as gene expression is an inherently stochastic process, studies involving small sample sizes are susceptible to a variety of concerns with regards to noise and the resulting uncertainty in observations. Several of these are discussed in further depth in S2 Text. Based on our analysis presented in S2 Text, we hold that for comparisons on the order of thousands of genes, *N* = 49 data points remains a reasonably safe minimum requirement.

Next, we remark upon the fact that *ρ*_*ij*,*k*_ and its sub-sample variation *σ*_*ij*,*k*_ are dependent: As a general rule, gene pairs with absolute correlations close to unity tend to exhibit smaller variation in co-expression between sub-samples. We present two possible explanations for this, one biological and one mathematical: If a large correlation for a pair of genes is important to cellular function, both gene products should be consistently present in nearly the same ratio in all of the sub-samples, e.g. through a process of tight gene regulation. Consequently, the observed variation in the co-expression pattern will be small. Strong correlations should therefore be more frequently associated with low variation between samples.

The mathematical explanation is based on the following observation: A large sub-sample variation *σ*_*ij*,*k*_ means that the sub-sample averages $r_{ij,k}^{l}$ must follow a broad distribution. However, it is impossible for sub-sample averages to be larger than unity. Thus, when *ρ*_*ij*,*k*_ is near unity, variation is limited in the sub-sample averages $r_{ij,k}^{l}$.

Finally, an important thing to keep in mind is that as the Spearman rank correlation is less accurate for small sample sizes, a choice of larger subsample size will generally bring the subsample correlations closer to the full-sample correlation. Consequently, *σ*_*ij*,*k*_ will generally be lower for higher subsample sizes. Because of this, it is important that the chosen sub-sample size is the same for both conditions studied in order to avoid an unbalanced contribution from one of the conditions to the denominator of Eqs 1–3.

### Gene relationship scores

In order to enable a systematic comparison of co-expressions, we introduce three pair-wise comparative gene co-expression scores which are computed for each pair of genes *i* and *j* in two different tissues. In general, these expressions may be applied to sets of data points from two different tissues, conditions or organisms:

$$C_{ij}=\frac{\left|\rho_{ij,1}+\rho_{ij,2}\right|}{\sqrt{\sigma_{ij,1}^{2}+\sigma_{ij,2}^{2}}},$$ (1)

$$S_{ij}=\frac{\left|\left|\rho_{ij,1}\right|-\left|\rho_{ij,2}\right|\right|}{\sqrt{\sigma_{ij,1}^{2}+\sigma_{ij,2}^{2}}}.$$ (2)

$$D_{ij}=\frac{\left|\rho_{ij,1}\right|+\left|\rho_{ij,2}\right|-\left|\rho_{ij,1}+\rho_{ij,2}\right|}{\sqrt{\sigma_{ij,1}^{2}+\sigma_{ij,2}^{2}}},$$ (3)

Here, *C*_*ij*_ quantifies the extent to which co-expressions for genes *i* and *j* are conserved, i.e. similar in both tissues. *S*_*ij*_ quantifies specific correlations: gene pairs which are strongly (positively or negatively) correlated in one tissue while showing no noticeable correlation in the other. Finally, *D*_*ij*_ describes the extent to which co-expressions are differentiated: *D*_*ij*_ is large for pairs of genes showing strong absolute correlations in both tissues or conditions, but where the nature of this correlation (positive or negative) changes between the two tissues. As the numerators for each of the expressions *C*_*ij*_, *S*_*ij*_ and *D*_*ij*_ are necessarily positive (being absolute values), and the denominator being a positive number potentially arbitrarily close to 0, *C*_*ij*_, *S*_*ij*_ and *D*_*ij*_ may assume any value from 0 (included) to infinity. However, as they follow widely different distributions, the three scores are not directly comparable within each other. In order to integrate the three types of co-expression into a common network, further steps are necessary in order to determine appropriate cut-off thresholds. We describe these in detail in the next section.

**Fig 1:**
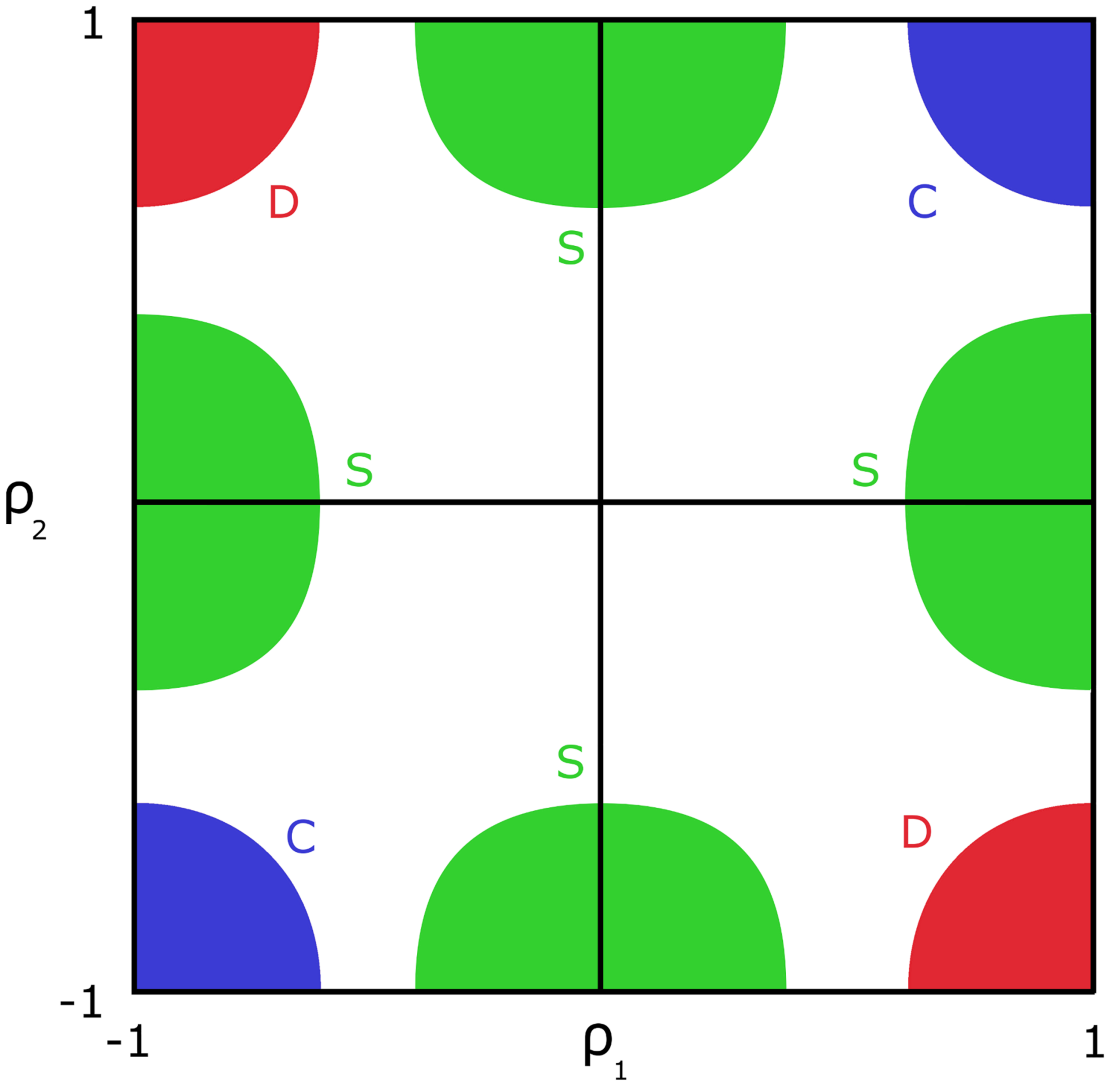
Gene co-expression score surfaces. General representation of the regions of interest for differential co-expression relationship scores C, S, and D. Here, *ρ*_1_ and *ρ*_2_ denote the Spearman rank-correlation of the expression of a given gene pair under condition 1 and condition 2, respectively. Colored regions correspond the three kinds of co-expression: blue is conserved C (strong co-expression in both conditions, no sign change), green is specific S (strong co-expression under only one condition), red is differentiated D (strong co-expression in both conditions, but with opposite signs). The colored letters indicate the scores associated with each colored region.

Fig 1 provides a schematic visual representation of the three co-expression patterns detected by our method. Our scores are designed in such a way that they assume large values within their respective areas (for instance, C-scores are large within the blue areas), while remaining small outside. Increasing the cut-off value for a given score is equivalent to shrinking the corresponding area of interest (restricting it near the corners for C and D, and along the middle of the edges for S). Since these areas converge on different points as the cut-off increases, a given gene pair may not exhibit large values for more than one score. Consequently, we can choose cut-off values for each score in order to uniquely classify relevant gene pairs according to the appropriate categories.

### Consolidated comparative gene co-expression network

In order to generate a network for the combined gene co-expression categories, the different ranges of the scores *C*_*ij*_, *S*_*ij*_ and *D*_*ij*_ necessitate a systematic approach for combining these interaction measures. For each of the three co-expression score types, we wish to determine suitable threshold values $k_{p}^{C,S,D}$ in such a way that these values correspond to the same importance level *p*. Thus, we will keep all gene co-expression scores $C_{ij}>k_{p}^{C}$, and discard those below this threshold. Similarly for *S*_*ij*_ and *D*_*ij*_ with their respective cut-offs. However, the three different interaction scores show distributions that have noticeably different means, medians, variances and general shapes (see S1 Fig). Consequently, determining whether pairs exhibit significant change or conservation based on either a common fixed-value cut-off or a given distance from the three means is incompatible with a meaningful comparison of significance across categories.

Instead, we determined the importance value of a random variable *X* based on the likelihood of obtaining said value from the underlying distribution: If the distribution is based on *M* data points (in our case, the number of different gene-pairs), we draw *m* samples *s*_*i*_ from the data set, and each *s*_*i*_ has the size *L* ≪ *M*. We determine the threshold value *X*_*p*_ as the average of the maximal values per sample:

$$\begin{array}{c} X_{p}=\frac{1}{m}\sum_{i=1}^{m}{\text{max}}_{\left\{s_{i}\right\}}X. \end{array}$$ (4)

The associated importance level is determined as *p* = 1/*L*. Thus, by choosing a common *p* for the gene relationship scores, we obtain a set of consistent cut-off values $X_{p}^{C,S,D}$ which we use to extract separate *C*-, *S*- and *D*-links that are combined in a final network. It should be noted that this *p* is not a significance threshold, as it is determined by the distribution of the scores for a given data set, not by testing the data against a null hypothesis. Instead, its purpose is to map the scores *C*_*ij*_, *S*_*ij*_ and *D*_*ij*_ on to a common scale, to allow for meaningful comparison between them.

### Measurement of node homogeneity

In the consolidated network, nodes may connect to their neighbors by either C, S or D link-type. In order to distinguish between nodes predominantly involved in one type of interaction and those with multiple different types of connections, we introduce the concept of node homogeneity *H*:

$$\begin{array}{c} H_{i}=\sum_{j\in \left\{C,S,D\right\}}{\left(\frac{k_{j,i}}{k_{i}}\right)}^{2}, \end{array}$$ (5)

using an expression introduced in a different context [38]. Here, *k*_*C*,*i*_, *k*_*S*,*i*_ and *k*_*D*,*i*_ denote node *i*’s number of C, S and D-type interactions, respectively, and *k*_*i*_ is the nodes degree (total number of connections). We note that in the extreme cases, *H* = 1 indicates a node with only one type of connections, while *H* = 1/3 (the lowest possible value) indicates a node with an even distribution of C, S and D-type connections.

### Disease association and KEGG pathway enrichment analysis

In order to determine enrichment of specific OMIM disease terms and KEGG pathways in our networks, we used Enrichr [39, 40] (http://amp.pharm.mssm.edu/Enrichr/) to obtain associated terms for each gene in the GTEx data set. This was a necessary step to establish accurate enrichment values, as Enrichr in itself does not provide for user-specified background gene lists. We then performed (Bonferroni-corrected) hypergeometric tests using NumPy (Python) for each of the 90 disease terms and 293 pathways listed in the Enrichr library to determine the significance of the number of associated genes in a given network.

### Software

In order to perform our analysis, we developed a set of software in-house, which has been made publicly available for download (https://github.com/andre-voigt/CSD). The code for computing Spearman correlations and variance was written in C++, with the remainder (computation of C, S, and D, estimation of cut-off, and network generation) implemented in Python. We used Cytoscape [41] to visualize the network, and NetworkX [42] to perform network analyses. External software used for the remaining analysis is listed in S3 Text.

Runtime, from expression data to the finished network may take anywhere from a few minutes to several hours for realistic data sets (scaling approximately quadratically with both the number of genes and the number of data points.). As an example, for a data set consisting of 1550 genes and 100 sample points per gene, complete run time is approximately 45 minutes on an Intel Xenon X5690 CPU.

## Results

### Comparison with existing differential co-expression methods

Table 1 provides a qualitative comparison between CSD and nine previously published methods for differential co-expression analysis that span a variety of method implementations. The defining characteristic of CSD is the classification of two types of differential co-expression (S-type; the loss of co-expression in one condition, and D-type; sign change) as well as the integration of conserved co-expression links in a composite network. Of the other listed methods, only the DCe method [33] recognizes S-type and D-type links as distinct forms of differential co-expression: it uses two different measures to determine the significance of co-expression change, one for same sign in both conditions and another when the sign changes between conditions. However, the DCe method does not distinguish between the two link-types in the final network analysis.

**Table 1:** Summary overview and characterization of differential co-expression methods. We characterize the presented methods by 5 tests: 1. Detects loss of co-expression. 2. Detects sign change. 3. Differentiates loss of co-expression from sign change. 4. Differentiates sign change and conservation. 5. Integrates conserved co-expression.

Method	Operating principle	Focus	1.	2.	3.	4.	5.	Main output
CSD	Direct score	Link	Yes	Yes	Yes	Yes	Yes	Full network
DCGL (DCe) [33]	Direct score	Link	Yes	Yes	Yes	Yes	No	Gene rankings, full differential network
DCGL (DCp) [33]	Direct score	Gene	Yes	Yes	No	Yes	No	Gene rankings
DiffCoEx [31]	Direct score	Link	Yes	Yes	No	Yes	No	Network modules
BMHT [34]	Direct score	Link	Yes	Yes	No	Yes	No	Network cliques, gene rankings
Choi (2005) [25]	Network comparison	Link	Yes	No	Yes	No	No	Full network, network clusters
Reverter (2006) [26]	Network comparison	Gene	Yes	No	Yes	No	No	Gene rankings
DICER [28]	Direct score	Link	Yes	Yes	No	Yes	No	Network modules
Gao (2013) [29]	Direct score	Link	Yes	Yes	No	Yes	No	Full differential network
DiffCorr [30]	Direct score, network comparison	Link, Module	Yes	Yes	No	Yes	No	Full differential network, differential clusters

The other listed methods either do not recognize the difference between D- and S-type links, or altogether omit D-type links from their differential co-expression analysis. Note that the CSD method explicitly includes conserved interactions in its resulting network, whereas C-type links are not included in the DCe method [33].

### Validation on simulated gene expression data

In order to critically validate our method’s ability to detect regulatory changes, we applied it to synthetic gene-expression data-sets with known, pre-defined regulatory interactions. As an initial test, we used a published synthetic gene data set from Zhang *et. al.* [43]. This has previously been used as a benchmark for relevant methods [27, 34]. This data set consists of 20 genes, and the two conditions are defined by changing 10 of the interactions, i.e. specifying 10 differentially expressed interactions. In particular, 5 of the interactions are present only in condition 1, and 5 (other) links are present only in condition 2. We recognize all of these 10 links as S-type links within our framework, and the specified network contains no D-type links. In S1 Table, we provide detailed results of our analysis, listing the 10 top-scoring gene pairs for C-type and S-type links, as well as the top 10 S-equivalent links found by DCGL along with a classification scheme for each pair. The relevant categories in this classification, based on their connections in the reference regulatory network, are as follows: direct C (immediate neighbors in both conditions), direct S (immediate neighbors in one condition only), indirect C (connected through one intermediary link in both condition), indirect S (connected through intermediary links specific to one condition). While other interaction schemes are possible, they do not occur among the top 10 links in either test.

Using our CSD-method to compute S-scores for all gene pairs, we find that the 10 direct differential interactions (DDIs) are assigned to 9 of the 10 top scores, with an indirect link [SWI4_SWI6, CLB6] incorrectly assigned the 8th place. The only DDI not making it into the top 10 is the [CLB6, MBP1_SWI6], scoring at 11th place. In contrast, the DCe method identifies 6 of the 10 DDIs amongst its top 10 scoring S-equivalent links, and with one non-differentially co-expressed link at the 10th place. DCe does identify 2 of the 10 DDIs ([MPB1_SWI5, CLB6] and [MBP1_SWI6, CLB6]) as “switched opposites” (equivalent to D-type in our terminology), but the last two selected links ([PHO2, CLB5] and [PHO2, CLB6]) are not identified as differentially co-expressed under standard parameters.

Similar testing of our method focusing on the conserved links identifies 4 of the direct conserved links among the top 10 C-scores, with the remainder consisting of genes separated by a single intermediary gene. However, we note that since correlations are transitive, it is entirely within reason that gene pairs indirectly linked in the regulatory network (but with strong co-expression along the intermediate steps) also show strong co-expression.

As this data set is unsuitable for thorough vetting of the CSD method due to the lack of D-type differential interactions, we used GeneNetWeaver [44, 45] to generate synthetic gene-expression data from networks containing both conserved, specific and differentiated links. Starting with a general regulatory reconstruction of *E. coli* (containing 1565 genes and 3758 edges) [44, 45], we randomly modified 10% of the interactions (5% removed, 5% switched from activator to repressor, or vice versa). We generated 200 synthetic gene-expression data-samples for both the original and the modified network. This process was repeated 20 times (generating new randomized networks and new synthetic expression data with 200 samples for *both* conditions each time), yielding 20 distinct sets of C, S and D-scores.

To assess the quality of our method’s ranking of links, we tested true-positive and false-positive rates for C, S and D-type interactions by comparing the ranked lists of C, S, and D-type scores for the links in the known test networks. We quantify the quality by receiver operating characteristics (ROC) curves. We also calculated ROC curves for the DCe method on the same networks and with the same synthetic gene-expression data as input. We used the DCGL package as a benchmark, as it is, to our knowledge, the only published method that is able to distinguish between S- and D-type links (and also demonstrates good performance in comparison to many existing methods [27]).

**Fig 2:**
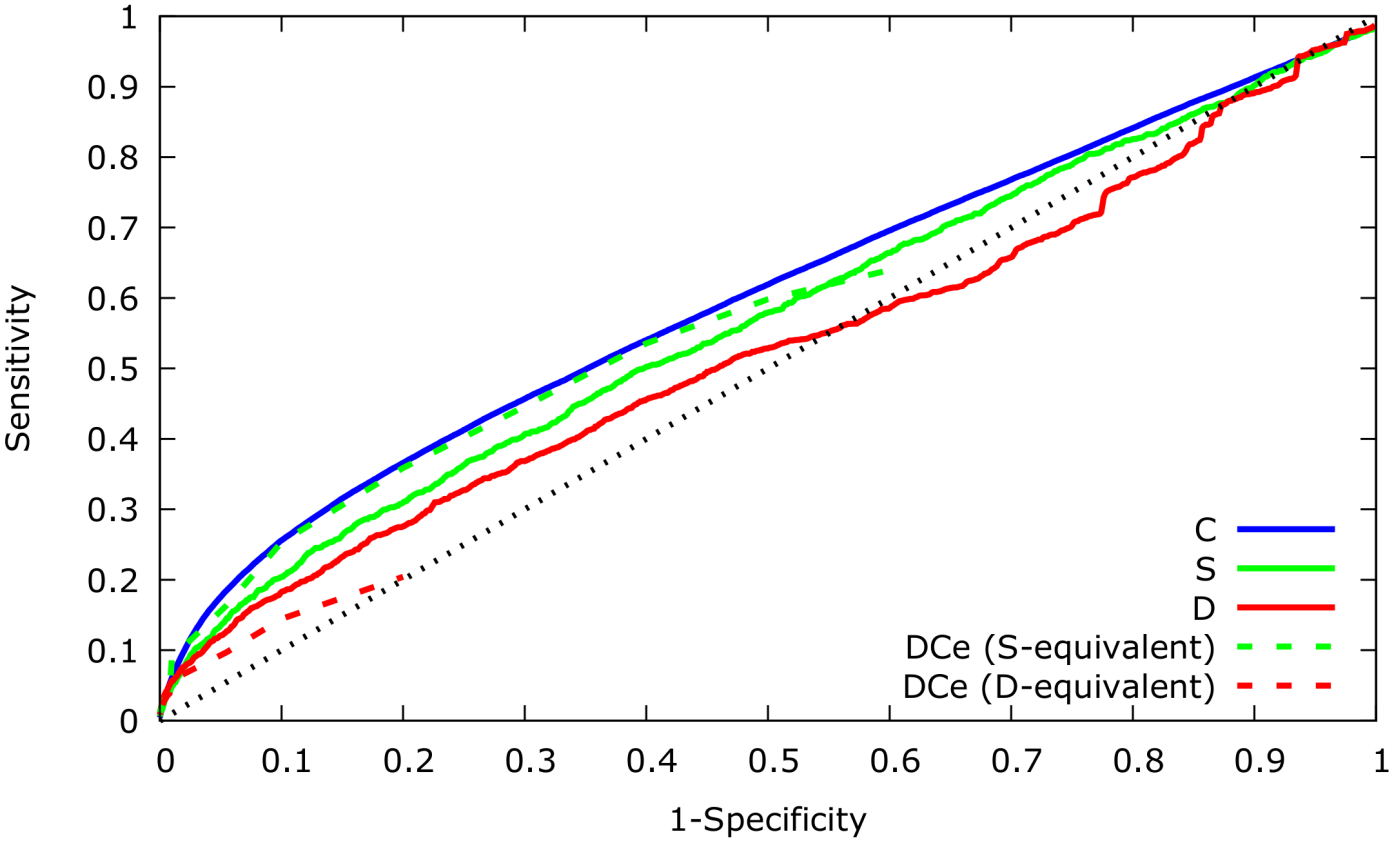
Receiver operating characteristic (ROC) curves for differential gene co-expression scores. ROC curves for the C, S and D-scores, and equivalent scores in the DCe method averaged over 20 independent simulations. The dashed black diagonal corresponds to *Sensitivity* = 1 − *Specificity*. Note that the DCe curves do not extend across the whole range, since DCe classifies genes detected as differentially co-expressed into either S-equivalent or D-equivalent, depending on sign change in the underlying correlation. Since a gene pair may only belong to one category in DCe, it is not possible to relax test requirements in such a way that one category contains all gene pairs. Notably, even under the most inclusive test requirements, the D-equivalent category can only contain on average ≈ 20% of gene pairs that show differently signed correlations between the two conditions.

Fig 2 shows the comparative ROC curves for the CSD and DCe methods. We find that on these data sets, CSD is substantially better at detecting D-type co-expression. However, despite the S-score’s success in identifying differentially expressed genes in the Zhang data set [43], it shows weaker performance than DCe on data generated in GeneNetWeaver [44, 45]. While we do not have any relevant method for which we can compare the performance of the C-score, we note that it’s general predictive power is higher than that of any of the other metrics.

The DCe method classifies genes detected as differentially co-expressed as either S-equivalent or D-equivalent, depending on sign change in the underlying correlation, with the consequence that DCe curves do not extend across the whole range in Fig 2: Since a gene pair may only belong to one category in DCe, it is not possible to relax test requirements in such a way that one category contains all gene pairs. Notably, even under the most inclusive test requirements, the D-equivalent category can only contain on average ≈ 20% of gene pairs that show differently signed correlations between the two conditions.

We observe that in general, the tested differential co-expression analysis methods do have substantial difficulties in accurately detecting individual regulatory perturbations for these types of network, and that even the best-performing measures in Fig 2 have a great deal of theoretical room for improvement in performance. This is generally the case for the existing methods [27]. A natural explanation for the difficulties in detecting individual changes lies in the fact that, empirical regulatory systems form complex networks, in which a given gene may be subjected to a multitude of regulatory impulses. Many of these input signals are shared with other genes, and regulatory cascades are common. Consequently, the loss of a regulatory interaction may not lead to a discernible change in co-expression correlations if these genes remain connected to shared regulators. On the other hand, an observed change in correlation between two genes may be the result of changes in regulatory mechanisms between intermediary genes in the regulatory network.

### Differential gene co-expression network in brain

**Fig 3:**
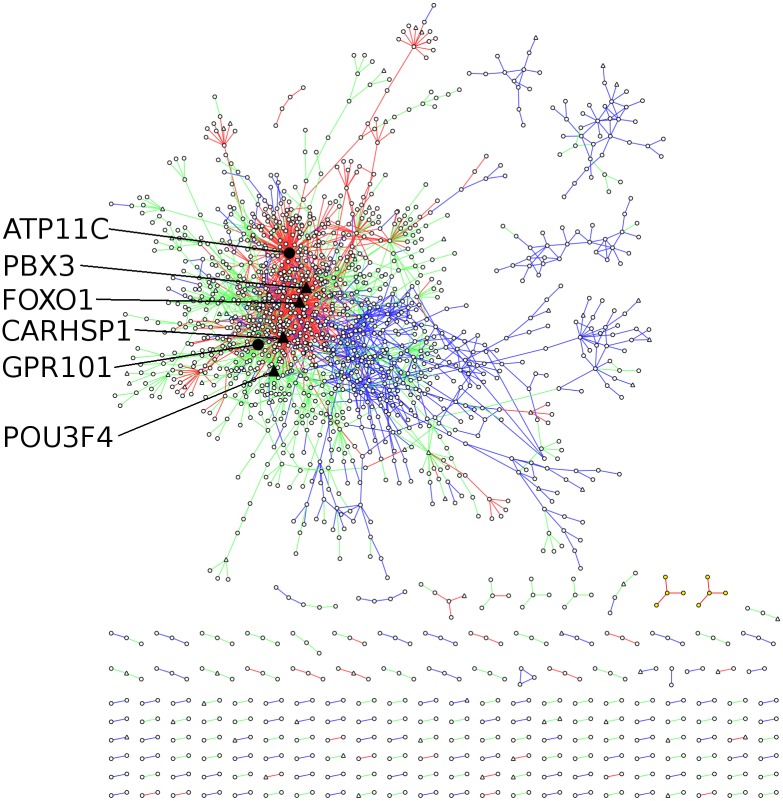
Overview of the CSD network. Visualization of the aggregate CSD-type network generated using a sample size of *L* = 10^5^. Triangular nodes indicate transcription factors. Prominent hubs (nodes with more than 40 neighbors) are colored black, enlarged and labeled for emphasis. Edges are colored by type: blue is C-type, green is S-type, red is D-type.

We selected the expression data from cortex and basal ganglia from the GTEx dataset to generate a CSD network. Using an importance level of *p* = 10^−5^ on the 18453 expressed genes, we obtained a network consisting of 1814 nodes (genes) and 2351 edges (Fig 3). Here, transcription-factor genes are indicated by triangle-node symbol. The network contains an even mix of edge types (767 are C-type, 806 are S-type, and 778 are D-type). A link to detailed data files describing the network can be found in S4 Text.

Fig 3 shows that the majority of the network is interconnected, forming a giant component consisting of 1333 (73.8%) nodes and 2024 (86.1%) edges. In addition to the giant component, we find 3 intermediately-sized connected components (respectively containing: 38 nodes and 47 edges, 30 nodes and 41 edges, 13 nodes and 13 edges). The remaining nodes form smaller connected components: 2 components of 5 nodes and 4 edges, 8 of 4 nodes and 3 edges, 28 triplets (all with 2 edges), and 137 isolated pairs. In Fig 3, we have highlighted the names and positions of the six genes with most connections in the networks. The top-3 list of most connected nodes consists of FOXO1 (*k* = 240 connections), ATP11C (*k* = 130 connections), and CARHSP1 (*k* = 120 connections), with a significant drop in connectivity to the fourth-most connected nodes (PBX3, *k* = 48).

A quick look at Fig 3 provides important insight concerning a key aspect of the network. It could be argued that, if conserved and differentiated interactions were functionally decoupled, and thus belonged to entirely separate parts of the genetic network, an integrated approach might not be particularly necessary, or even useful. In contrast, we find a highly interconnected network, with core regions densely interconnected by all three types of interactions. However, while the different interaction classes do not form separate networks, there is a distinct tendency for links with the same score type (either being C, S or D) to group together.

**Fig 4:**
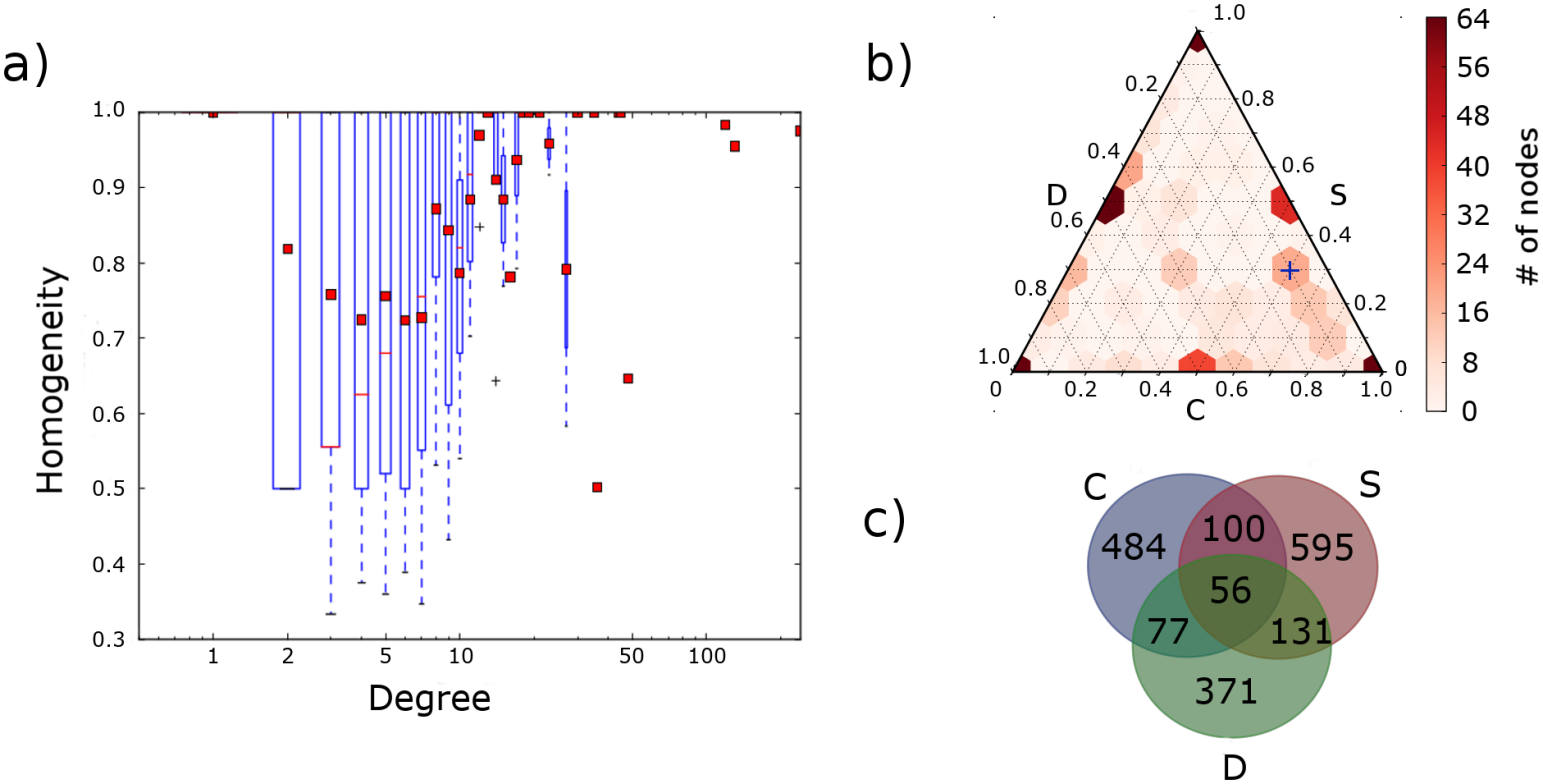
Node homogeneity and mixing of interactions. a) Box plot of gene homogeneity scores *H* according to node degree. Red bars denote the median *H* for nodes of the specified degree, and red squares denote the mean. Bottom and top ends of the boxes represent the first and third quartiles, respectively. The end of the whiskers correspond to min/max values of *H* at that degree. b) Ternary heatmap, detailing the fractions of specified interactions *k*_*j,i*_/*k*_*i*_ with *j* ∈ {*C*, *S*, *D*} per gene: Corners correspond to homogeneous nodes, i.e. nodes with only one type of interaction. The sides correspond to nodes with two types of interactions (scale is fraction × 10), e.g. *k*_*C*_ = 0 along the side marked D. The blue cross is an aid, with coordinates (*C* ∼ 60%, *S* ∼ 30%, *D* ∼ 10%. c) Venn diagram showing the relative quantities of genes involved in each type of interaction.

We investigated the propensity of nodes to be connected with links of different types by calculating the homogeneity-score H (Eq (5)) for each node. Fig 4(a) shows a box-plot of H as function of degree. Of the 1333 nodes in the giant component, 333 (just short of half of the 701 nodes with at least 2 neighbors) have interactions of at least two of the three different types, and 56 (approximately 1 in 8 of the 404 nodes with at least 3 neighbors) have interactions of all three types (Fig 4(c)). Interestingly, Fig 4(a) suggests that highly connected genes are dominated by specific types of interactions, as shown in Table 2. Here, of the top 5 hubs for each category, all but one of the C-hubs and one of the D-hubs have homogeneity scores over 0.9. On the other end of the degree distribution, we note that nodes with very few (less than 4) neighbors also tend to have somewhat more homogeneous neighborhoods than nodes with intermediate connectivity.

**Table 2:** Network hubs for each type of interaction. *k* denotes node degree (total number of connections), while *k*_*C*_, *k*_*S*_ and *k*_*D*_ denote the number of connections of each type (*k*_*C*_ + *k*_*S*_ + *k*_*D*_ = *k*). *H* denotes node homogeneity, as defined in Eq 5.

Top 5 C	*k*	*k*_*C*_	*k*_*S*_	*k*_*D*_	H
UBQLN1 TRIM37 FAXC PPP3CB ATP6V1C1	23 17 15 14 15	22 17 15 14 13	1 0 0 0 2	0 0 0 0 0	0.92 1 1 1 0.77
Top 5 S	*k*	*k*_*C*_	*k*_*S*_	*k*_*D*_	H
GPR101 POU3F4 ECE2 DESI2 DIRAS3	45 44 35 30 30	0 0 0 0 0	45 44 35 30 27	0 0 0 0 0	1.0 1.0 1.0 1.0 1.0
Top 5 D	*k*	*k*_*C*_	*k*_*S*_	*k*_*D*_	H
FOXO1 ATP11C CARHSP1 PBX3 DDO	240 130 120 48 21	0 0 0 0 0	3 3 2 11 0	237 127 118 37 21	0.98 0.95 0.98 0.64 1

Obviously, for nodes with only one neighbor, *H* = 1, while for nodes with only two neighbors, *H* must be equal to either 1 or 0.5 (whereas the lower bound on *H* for *k* ≥ 3 is 1/3).

If we classify any gene with 3 ≤ *k* ≤ 10 as an intermediate gene, and genes with *k* > 10 as a hub, we find that intermediate genes are significantly less homogeneous than hubs (t-test, *p* = 3.8 ⋅ 10^−5^). Looking over the combined set of intermediate and hub genes, higher degree nodes are positively correlated with increased homogeneity (Spearman *ρ* = 0.082, *p* = 0.085; for all genes with *k* ≥ 4, *ρ* = 0.2, *p* = 8.2 ⋅ 10^−4^).

How are the different link-types spread among the nodes? Fig 4(b) is a ternary heatmap showing a histogram of the three fractions *k*_*j*,*i*_/*k*_*i*_, with *j* ∈ {*C*, *S*, *D*} for each node. Consequently, entries at the corners account for all nodes with *H* = 1 (see panel (a)), whereas entries at either of the sides correspond to the nodes of Fig 3 connected with only two kinds of links. For entries in the interior, the corresponding nodes are connected to all three kinds of links. For a given point on the triangle, the corresponding proportion of interactions of type *X* is determined by following the line parallel to the base *X* = 0 until reaching the base labeled *X*. To illustrate this, we have provided an example by marking the tile corresponding to a mix of approximately 60% C, 30% S and 10% D (density of 19 nodes) by a blue cross. As the densities at the corners (representing nodes containing only one type of interaction) are far higher than anywhere else, the color scale has been truncated at the highest non-corner value (0% C, 50% D, 50% S, 64 nodes). Whereas the Venn-diagram (Fig 4(c)) details the number of nodes with a given mixture of links, the ternary heatmap shows how the links are mixed at the nodes. Panel (b) shows that the majority of the nodes connected to the three link types are dominated by C-specific (fractions above 0.6), and some S-specific (near 0.3), but only with a few D-specific interactions (fraction near 0.1).

### Robustness and features of interaction network to choice of *k*_*p*_

**Fig 5:**
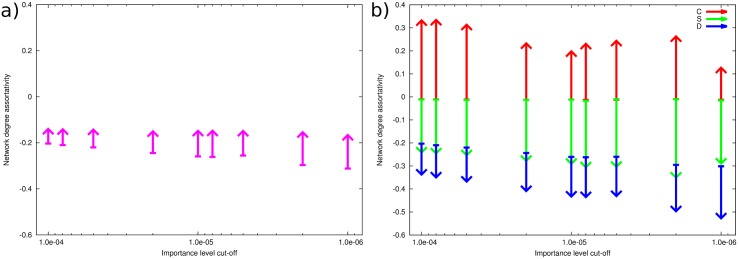
Robustness of network topology. a) Degree assortativity for the consolidated comparative gene co-expression network, generated for different importance levels. Lines denote the difference between maximum k-core in the empirical network at the selected threshold (arrow tip) and mean degree assortativity across 100 random networks with degree distributions similar to the empirical network (arrow tail). b) Similar to a), but for networks consisting of interactions of each individual type (C, S, and D). Empirical C-networks show positive assortativity as well as higher than random k-core, indicating an affinity between tightly connected nodes and a “rich club”-structure, while the empirical S- and D-networks show negative assortativity and lower than random k-core, indicating a hub-and-spoke type network structure.

We evaluated the consequence of different cut-off values *k*_*p*_ for the structure of interaction specific networks by generating separate *C*-, *S*- and *D*-networks for a range of importance values *p* ∈ [10^−6^, 10^−4^]. For each interaction type network, we calculated their degree distribution, degree assortativity and max *k*-core number, and identified the top-10 most connected genes in each network. Fig 5 shows that while the *C*-networks exhibit greater positive degree assortativity than randomized networks with the same degree distribution, S and D networks are disassortative with respect to degree. We also find that the C-type network exhibits a higher maximum k-core value than randomized networks at the same degree distribution, while S- and D generally exhibit lower maximum k-cores (S6 Fig). Both of these traits indicate that C-networks are dominated by reasonably densely interconnected sets of genes of the same type (with highly connected genes generally connecting to other highly connecting genes, and sparsely connected genes generally connecting to sparsely connected genes), while the S- and D-networks follow a hub-and-spoke topology, where certain prominent genes connect to a large number of neighbors, which themselves connect to one or a few prominent nodes.

We have verified that the selection of a specific cutoff in the indicated range has little effect on the topology of the resulting network, and in the case of S and D, the relative ranking of prominent hubs remains relatively constant (S2 Table). Due to the comparatively small differences in connectivity of main hubs in the C-network, the changes in node rank are more pronounced, although the relative connectivity (node degree relative to the most-connected hub) remains stable. This allows us to select cutoffs for C, S and D to yield reasonably tractable networks, while ensuring that the specific cutoff chosen does not have a dramatic effect on key aspects of the resulting network and our presented analysis.

We note that the topological differences between the C, S, and D-networks revolve around three main characteristics: degree distribution, assortativity and clustering (defined as the proportion of common neighbors between two directly linked genes). In general terms, the C-network is tightly knit, with high clustering and a rather narrow degree distribution (with less prominent hubs). The D-network is opposite, where most D-links connect high-degree (*k* > 20) genes with otherwise isolated or near-isolated genes *k* ≤ 3. Additionally, the clustering coefficient in the D-network is zero, and no genes that are directly connected to each other by a D-link are also connect to a common neighbor through other D-links. The S-network lies somewhere in between these two characteristics: its hubs are more prominent than those of the D-network, but less than those of the C-network, and its clustering coefficient is also somewhere in between.

The differences in clustering are the result of mathematical factors—specifically, the transitivity of strong correlations. Considering three genes *i*, *j* and *k*, it follows from the nature of correlations that if |*ρ*_*ij*_| ≈ |*ρ*_*ik*_| ≈ 1, then *ρ*_*jk*_ ≈ *ρ*_*ij*_*ρ*_*ik*_. Because of this, if *ρ*_*ij*_ and *ρ*_*ik*_ remain strong and constant between conditions, then so must *ρ*_*ij*_, naturally creating “triangles” in the C-network. Assortativity is also a natural consequence of this—if gene *i* is strongly correlated with gene *j*, then *j* will generally tend to be correlated with *i*’s neighbors—therefore, if *i* has many neighbors, *j* is likely to have many neighbors as well.

Similarily, should *ρ*_*ij*_ and *ρ*_*ik*_ simultaneously switch signs, it is mathematically not possible that *ρ*_*jk*_ also switches signs, as three genes may not all be strongly negatively correlated with each other. In fact, the D-network can be approximately characterized as a so-called bipartite network (ignoring any potential weakening of the transitive effect over longer distances and weaker links). A bipartite network is defined as a network in which each node can be categorized into either of two groups, and where there are no direct links between two nodes belonging to the same group. As a direct result, a bipartite network cannot contain closed triads, and therefore has a clustering coefficient of 0.

In the case of the D-network, however, transitivity of correlations does not, by itself, adequately explain the extreme disassortativity we observe. In as much as the D-network necessarily forms a bipartite network, we noticed that the two characteristic groups roughly correspond to hubs and non-hubs. This is not a mathematical necessity—one can readily find bipartite networks in which the majority of direct connections are between hubs (or between non-hubs), or with a very narrow degree distribution. We could, for instance, create a co-expression network consisting of a giant component divided into two groups of equal size, and each node connects to each of the nodes in the other groups; we could then add any number of isolated connected gene pairs. This would constitute a bipartite network consistent with a correlation network, but highly assortative.

A possible explanation for the disassortativity of the D-network could reside in an argument from parsimony—that the underlying regulatory switches would happen at the individual gene level, that these are reasonably rare, and that changes to one or a few genes in a cluster would not substantially affect the relationship between the other genes in that cluster. In this case, the few perturbed genes would show D-type connections to the majority of the genes that remained constant, while the unperturbed genes would connect only to the few perturbed genes.

### Functional enrichment

In order to establish whether the observed network relates to possible functional aspects of the invetigated tissues, we performed GO biological process enrichment analysis using GOrilla [46, 47] (http://cbl-gorilla.cs.technion.ac.il/) on 4 networks: separately for the C, S and D-type networks generated with a draw size of 10^5^ pairs, as well as the combined network obtained by merging the individual C, S, and D-networks.

For each of the 4 networks, we found significant enrichment for a variety of biological processes (S2, S3 and S4 Figs). In all cases processes related to nervous functions are enriched, ranging from specific concepts (e.g. regulation of neuron projection development) to general ones (cognition, behavior). Of these, GO categories for ‘anterograde trans-synaptic signaling’ is particularly prominent, showing highly significant enrichment in each of the 4 networks. It is reassuring for our method to find these GO categories as being over-represented, since we analyzed data from brain tissues.

Among the remaining enriched terms, we mainly find processes related to cellular differentiation and localization, metabolism, transport and signaling. While these processes are not important for brain functions only, their enrichment in the network seems far from surprising in a co-expression network of brain tissues, given the exceptional energy requirements of the brain.

### PBX3: A variable-connection hub

While most prominent hubs in our network tend to connect to their neighbors through only one type of edge, a few genes exhibit a substantial number of connections of different types (see Fig 4). The most prominent of these is the transcription factor PBX3. In developing macaque brains, PBX3 expression is upregulated in the basal ganglia and the cerebral cortex, suggesting a possible role in brain development [48]. However, PBX3 is mostly known as an oncogene involved in a variety of cancer types. One of these is pilocytic astrocytoma [49] (PA)—a form of glioma most commonly occurring in the cerebellum or areas near the brainstem (which include the basal ganglia), but not in the cerebral cortex [50], and more frequent among children and young adults [49]. The fact that our network analysis points out PBX3 as a hub with connections of different types, might hint at a molecular explanation for the differential occurrence of PA in these two tissues we analyzed.

Looking at PBX3’s neighborhood, we find several other genes with similar characteristics. First, all connections this network represents are strongly positive correlations in basal ganglia. Accordingly, S-type connections correspond to weak absolute correlations in the cortex, while D-type connections correspond to strong negative correlations in the cortex. Out of 48 neighbors, 8 are suspected of influencing the development of glioma. Of these 8 genes, 6 (SULT4A1 [51], NDRG4 [52, 53], GAP43 [54, 55], BEX1 [56], HINT1 [57], LZTS1 [58]) are believed to act as tumor suppressants, while the remaining two, PKM [59] and VIPR1 [60–62], have been found to be overexpressed in glioma. Several of these genes also appear to play an important role in mammalian brain development and cell differentiation, where the genes VIPR1 [63], NDRG4 [64, 65], BEX1 [66] and GAP43 [67] have been found to exhibit increased expression in the brain of young rats or monkeys.

### KEGG pathway enrichment

Using the 2016 KEGG Pathway database through Enrichr [39, 40] (http://amp.pharm.mssm.edu/Enrichr/), we searched our network for overrepresented terms. Detailed results are provided in S3 Table. The whole network shows significant enrichment for categories including dopaminergic synapse (S, D), oxytocin signaling (S, D), adrenergic signaling (D), glutamatergic synapse (D), endocannabinoid signaling (D) and GABAergic synapse (D). The C-network shows fewer significantly enriched pathways—the most prominent being the synaptic vesicle cycle pathway.

We note that the most enriched pathways revolve around chemical compounds well known for their role on the nervous system. This is not unexpected, as our data come from two types of brain tissue. Interestingly, these pathways are not particularly well-represented in the C-network, but are ubiquitous in both the S- and D-networks. This might indicate that while these compounds play important roles across the nervous system, there might be significant regulatory differences between different types of brain tissue.

### Relation to protein interaction networks

In an effort to find possible causal links behind observed CSD-links, we searched the human protein interaction network (PIN) for connections between nodes in our network. Since CSD-links are based on co-expression analyses, it is not a given that these (often) indirect relationships should be reflected in direct interactions in the PIN. However, as protein-protein interactions are functionally dependent on both proteins being expressed simultaneously, we would expect these to be a potential source of C-type interactions. The PIN used for the search was compiled from three sources: the Center for Cancer System Biology’s human interactome project (HI-II-14) [68], CCSB’s literature data set (Lit-BM-13) [68], and BioGRID [69]. As the BioGRID data set is not particularly stringent when including an interaction, we decided to only include BioGRID interactions backed by at least two sources. The resulting combined PIN contains 49972 interactions for 10349 genes. 9417 of these genes are also present in the original GTEx expression data—approximately 51% of the total number of genes in the GTEx data set. Of the 1798 genes present in the combined CSD network, 1063 (59%) are connected to at least one other gene in the combined PIN. This shows a moderate over-representation (factor 1.16, *p* < 10^−6^) of PIN genes in the CSD-network.

Interestingly, 7 gene pairs are directly linked by edges in both the PIN and the CSD network (see Table 3). While this is a small section of either network, it is still a substantially larger overlap than would be expected by random chance: comparing 10^5^ randomized versions of the CSD network (each made by random selection of 1798 genes and 2351 gene pairs from the 18453 genes in the GTEx data) with the PIN as the null case, we find an expectancy of edge overlap on average to be ≈ 0.4, with a single case of 6 overlapping edges as the maximum observed overlap. Accordingly, the observed overlap between the actual CSD network and the PIN is approximately 18.7 times greater than the null hypothesis (*p* < 10^−5^). Further, we note that of these overlapping pairs, 6 are C-type edges in the CSD network (the last pair being D-type).

**Table 3:** Gene pairs directly connected in both the PPI and CSD networks.

Gene A	Gene B	Type of CSD interaction
C1QA	C1QB	C
CARHSP1	PNMA1	D
CD74	HLA-DRA	C
HCK	WAS	C
HERC3	UBQLN2	C
RPS11	RPS3	C
S100A8	S100A9	C

In order to investigate more indirect links, we also computed shortest paths across the PIN for each pair of nodes directly connected in the CSD-network, in order to establish whether other CSD-type connections could relate to protein interactions. We found that the genes in the differential co-expression network are more closely connected to each other than average in the PIN, with an average path distance of 3.95 (against 4.04 for the whole network). While the magnitude of this effect is small, it is highly significant with *p* ≪ 10^−3^ and *z* = 6.29 (based on the standard deviation of the mean distance of similarly sized random samples of the whole PIN). This suggests that the protein-protein interactions may explain certain connections in the CSD, although they are most likely not the main factor.

In order to find possibly relevant mediating genes, we sorted the nodes in our PIN according to the number of shortest paths (between genes directly connected in the CSD network) they appeared in, with the added caveat that those paths consisted of at most 3 steps (meaning there could be at most 2 intermediate genes in the PIN). The purpose of the 3-step limit being to eliminate highly indirect connections in the PIN, which are less likely to reflect an actual functional relationship.

The most prominent intermediate genes in the PIN network include ESR1, AKT1, MDM2, TRAF1, UBE2I, SIRT1 and PPP1CA. Most of these genes are known to be involved in processes which should be relevant to the differentiation and function of neural tissue, such as regulation of gene expression (ESR1, AKT1, UBE2I, SIRT1, PPP1CA) and metabolic/catabolic processes (AKT1, MDM2, UBE2I, SIRT1, SKP2, PPP1CA). In more specific detail, ESR1 and TRAF1 are both involved in regulation of NF-kB signaling—ESR1 as an inhibitor and TRAF1 as an activator. NF-kB is known to be involved in synaptic plasticity, learning, and memory, and may be activated by synaptic transmission. Promoter hypermethylation at ESR1 [70], expression of TRAF1 [71] and mutations in NF-kB [72] are all known to be associated with the emergence of glioma. AKT1 is known to interact with forkhead box transcription factors [73] (which include FOXO1, the most highly connected node in the differential co-expression network) in order to regulate cell growth and apoptosis. As FOXO1 connects to several of the cancer-associated genes adjacent to PBX3 (though not to PBX3 directly), the relative prominence of both FOXO1 and AKT1 might reflect a potential tumor-inhibiting effect in combination with PBX3 and its neighbors.

### Disease association

As we were able to identify a number of key neurological process amongst the genes present in our networks, we sought to investigate if there could be any links between differential co-expression and inheritable disease. Using the extended OMIM disease association data set, we found no significant enrichment for disease-associated genes in general in the combined network (*p* = 0.417) or in the C-only or D-only networks (*p* = 0.175 and *p* = 0.649, respectively). However, we did find an over-representation (by a factor of 1.35) of disease-associated genes amongst genes in the S-network (non-corrected *p* = 0.00458). Using Enrichr [39, 40] (http://amp.pharm.mssm.edu/Enrichr/) to search the C, S, D- and combined networks for specific OMIM disease associations, we find substantial enrichment for one of two disease families, depending on the kind of network. The S-network shows an over-representation of genes associated to epilepsy, with 8 genes in the network, while only 1.7 genes would be expected by chance (4.65-fold enrichment, *p* < 0.4 ⋅ 10^−3^). The D-network shows enrichment for ataxia, with 6 genes (expected number 1.1, 5.4-fold enrichment, *p* < 8 ⋅ 10^−3^), and more specifically, spinocerebellar ataxia, with 5 genes (expected number, 0.62, 8-fold enrichment, *p* < 2 ⋅ 10^−^3). While both terms are rather broad and may refer to any of a variety of diseases with different underlying mechanisms, they both involve defects in motor functions, which are controlled by basal ganglia and cerebellum.

**Fig 6:**
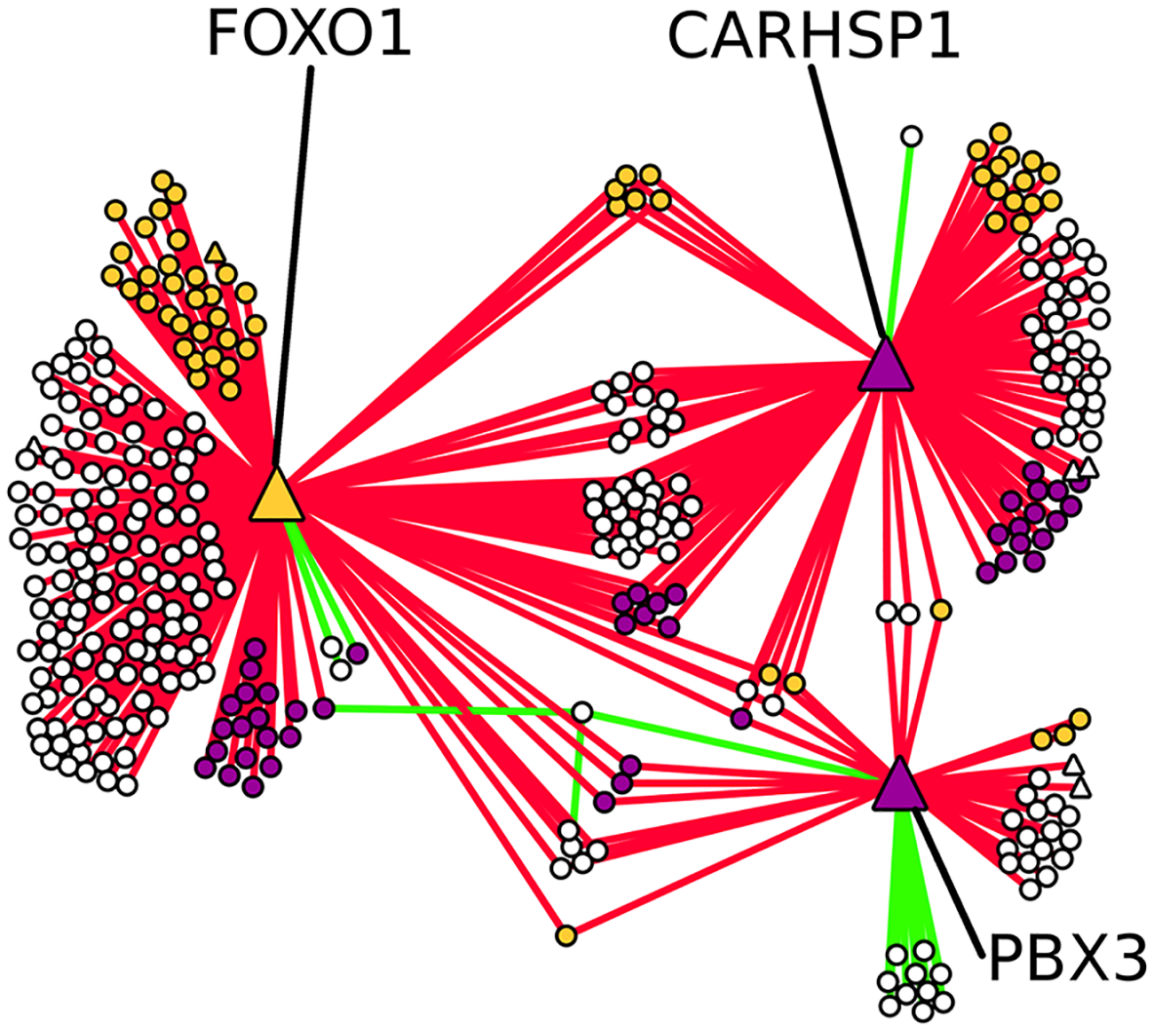
Network hubs and glioma associations. Neighborhood of the glioma-associated hubs FOXO1, CARHSP1 and PBX3. Every represented gene connects to at least one of the hubs. Non-hub genes are grouped according to the hubs they connect to (and by interaction type), as well as regulation in glioma. Transcription factors are denoted by triangular labels, other genes with circles. Purple nodes represent genes whose activity is positively linked to harmful outcomes in glioma, while the activity of yellow node is linked to more benign outcomes. White nodes represent genes without established links between activity and glioma. Red links are D-type connections, while green ones are S-type. There are no D-type connections linking two non-hub genes to each other.

Noting that three of the four dominant hubs exhibit protein interactions with a number of glioma-related genes, as well as the presence of several glioma-related genes in PBX3’s neighborhood, we mapped out their immediate network (see Fig 6). Furthermore, we performed an exhaustive literature search to identify whether any of FOXO1 or CARHSP1’s immediate neighbors also exhibited particular expression patterns related to glioma. In fact, in the combined neighborhoods of FOXO1, CARHSP1 and PBX3, we find 104 such genes (out of a total of 340 in said neighborhoods) (see Table 4 for a detailed listing). For most of these genes (59), increased expression is associated with beneficial outcomes, while 45 genes have their activity linked to increased proliferation, invasiveness and general mortality. The hubs themselves are all associated with aggressive forms of glioma. As previously mentioned, PBX3 is known to be upregulated in PA [49], while increased CARHSP1 expression is linked to necrosis and microvascular proliferation (MVP) [74]. On the other hand, FOXO1 is known to prevent cell proliferation in glioblastoma [75].

**Table 4:** Glioma-associated nodes in the neighborhood of FOXO1, CARHSP and PBX3. Associations between gene activity and glioma (as found in literature) are divided into two general groups: Positive associations denote any gene where increased expression is generally linked to harmful outcomes for the patient. This includes genes which are overexpressed in glioma as opposed to healthy tissue, where increased expression in glioma is correlated with higher mortality, or where the gene is more highly expressed in higher grade gliomas. Negative associations denote genes where higher expression is generally linked to beneficial outcomes for the patient.

Neighboring hub(s)	Positive	Negative
FOXO1	MBTPS1 [76], RBP4 [77], HN1 [78], SSTR2 [79], LNX1 [80], ENO2 [81], CDK5 [82], ABCC1 [83], EFNB3 [84], SMYD3 [85], HABP4 [86], PFKM [87], ITPR1 [88], NNAT [89], CYFIP2 [90], ARG2 [91], ARF3 [92], HSPA12A [93], MYO10 [94],	DCTN2 [95], ACTR3B [96], MTCH1 [97], SLC1A6 [98], NBEA [99], MAP2K4 [100], TNPO2 [101], MOAP1 [102], ARPP21 [103], CADM3 [104], KCNA4 [105], SVOP [106], REPS2 [107], SLIT2 [108], PANX1 [109], CCT7 [110], KCNMA1 [111], CACNA2D3 [112], KCNV1 [113], PIK3CB [114], NPTX1 [98], CDH18 [115], GLS2 [116], NRIP3 [98], TACC2 [117], CALM3 [118], NELL2 [119], CBX7 [120], MTA3 [121], AJAP1 [122, 123], PARK2 [124], PI4KA [125]
CARHSP1	MADD [126], STXBP1 [127], TMX4 [128], GLS [116], KCNK3 [129], MAP2 [130], YWHAB [131], PANK2 [132], UCK2 [133], ATXN10 [134], ATP1A1 [135], EPB41L4B [136], DRP2 [137], CALM1 [138], CHAF1B [139]	ST6GALNAC5 [140], OLFM3 [141], PCDH8 [142], PRPF19 [143], CHGB [144], DUSP4 [145], SLC32A1 [146], PPP1R14C [147], MACROD2 [148], ATP6V1B2 [149], YWHAH [97], CERS6 [150], SCG2 [151], GRM7 [152]
PBX3		BEX1 [56], HINT1 [57], LZTS1 [58]
FOXO1 + CARHSP1	PAK1 [153], PPME1 [154], PGK1 [155], UCHL1 [156], SYNGR1 [157], MAP2K5 [158], TPI1 [159]	RNF41 [160], RTN1 [123, 161], SGSM1 [162], SGSM1 [162], BTBD10 [163], DLG3 [164]
FOXO1 + PBX3	VIPR1 [60–62], CCKBR [165], PKM [59]	SNRPN [166]
CARHSP1 + PBX3		SULT4A1 [51]
FOXO1 + CARHSP1 + PBX3	FBXO16 [167]	NDRG4 [52, 53], GAP43 [54, 55]

We also note that the gene-glioma associations presented come from a variety of previously performed studies, and that there is no guarantee that the literature contains an exhaustive list of genes involved in glioma. It is therefore quite possible that there are genes important to glioma development whose role has not yet been discovered, and consequently, would not have been identified here. The substantial presence of known glioma-associated genes in the neighborhood of FOXO1, PBX3 and CARHSP1 may indicate the additional presence of genes with currently unidentified roles in glioma. We therefore present the exhaustive neighborhoods of FOXO1, PBX3 and CARHSP in S4 Table Text as candidate genes for further study.

## Discussion

In this paper, we describe a new method for identifying differential co-expression relationships between genes when comparing two tissues. In contrast to previous methods, our method allows the detection of genes that play critical roles in context-specific function, based on similarities and differences in co-expression patterns. We demonstrate the power of our new method by analyzing the network of cortex and basal ganglia tissues, which is revealed to be associated with a variety of important aspects of brain function. In particular, we find substantial enrichment of (1) GO terms such as anterograde synaptic signaling, cognition, and neural development, (2) hereditary links to the neurological diseases ataxia and epilepsy, and (3) genes associated with pathways involving compounds important to brain function, such as adrenaline, dopamine and oxytocin.

Furthermore, we find indications for the hub PBX3 to be involved in the occurrence of PA—which occasionally occurs in basal ganglia but not in the cortex. In addition, we suggest that the general preponderance of GO terms with clear relevance to brain development and function indicates that the resulting network represents genuine and meaningful relationships between the genes present in the network.

While the gene expression data used in this study came from the same source, this is not a requirement for the method to be viable. Since the networks are based on the non-parametric Spearman rank-correlation (which relies only on the relative rank of each data point within its set) calculated within each of the compared data sets, it is not necessary for the expression values in the different sets to be normalized against each other. In fact, one could compare a tissue with log-scale expression values (e.g. coming from microarrays) against one where the expression values follow a linear scale (e.g. RNA-Seq data), without any impact on the resulting network.

It should be noted, however, that the networks obtained by this method do not correspond to protein-protein interaction networks or even gene regulatory networks, and that the presence of a link between two genes in the differential co-expression network does not necessarily reflect any direct biological interaction between the two. In fact, a link is only evidence of a coinciding pattern: two co-expressed genes may both be regulated by a common transcription factor, or may be similarly affected by outside factors (for instance, nutrient availability).

We note that triple-type nodes (involved in all three types of interactions) are dominated by C-type interactions and a very small share of D-type interactions. We also note that the leading D-type hubs have far more connections than those of the other types. This may suggest that the D-type regulatory change between tissues demonstrates a much more concentrated effect: even if the underlying changes are focused near only a few key genes, a disproportionately large amount of interacting genes could be affected.

The key topological difference between the C-type network on one hand (highly assortative and with a substantial densely connected core) and the S- and D-type networks on the other (with a few dominant hubs, especially in the case of D) also indicate a possible difference regarding the regulatory mechanisms involved. Hence, we speculate that a tightly co-regulated cluster of genes might involve more redundant (and thereby robust) regulatory mechanisms and therefore be less likely to change. In contrast, genes with more centralized neighborhoods may be more likely to see large changes in co-expression patterns due to perturbations at the individual gene levels. An alternative hypothesis is that the strong prominence of hubs in the D-network comes as a result of regulatory changes mostly involving a few genes within large co-expressed clusters, whereby the few perturbed genes would form D-type links with the remainder of said clusters.

We take particular note of a set of gene clusters, centered around the transcription factors FOXO1, CARHSP1 and PBX3. These consist of multiple genes believed to be of major importance to both neural development and the emergence of glioma. While it is known that defects in genes controlling growth and differentiation is a common factor in cancers, to the best of the authors’ knowledge, no association between these specific genes has previously been determined. However, it is difficult to present conclusions about the underlying cause of the observed co-expression patterns as certain. While the FOXO1/PBX3/CARHSP1-centered gene clusters suggest a functional link between several glioma-associated genes, it does not, for instance, automatically follow that misregulation of (or by) PBX3 is the key driver in glioma development.

While it is hard to determine a definite cause behind these connections at the gene level, a comparative study between the CSD network and the PIN offers one possible explanation. We find that in the PIN, both PBX3 and CARHSP1 are indirectly connected to each other (as well as several of their other neighbors in the CSD-network) through the intermediary of TRAF1, whose overexpression is also associated with the emergence of glioma [71]. We also find similar intermediary protein interactions through ESR1, AKT1 and SIRT1, whose activity are also associated with glioma [70, 168, 169]. Additionally, AKT1 is known to interact with FOXO1 (the most prominent hub in the CSD network) to inhibit apoptosis [73]. FOXO1 and AKT1, along with MDM2 (another common intermediary gene in the PIN) have previously been identified in differential co-expression studies of glioblastoma [170].

The prominence of glioma-related cells in these clusters is somewhat unexpected, as our comparison is not between cancerous and non-cancerous data sets, but rather of two (nominally healthy) different parts of the brain. However, we note a substantial overlap between glioma-associated genes and genes particularly expressed in developing (embryonic and juvenile) brains. Additionally, GO enrichment tests using the Gene Ontology Consortium database (www.geneontology.org) [171] return an 1.8-fold enrichment (Bonferroni-corrected *p* = 2.1 ⋅ 10^−2^) for the term “nervous system development” amongst the 340 genes in the neighborhood of FOXO1/CARHSP1/PBX3, and a 2.7-fold enrichment (corrected *p* = 1.2 ⋅ 10^−3^) for the same term among the 104 genes for which we found associations with glioma. The latter 104-gene set also shows significant enrichment for more specific subterms of “nervous system development”, including “neuron differentiation” (3.5-fold, *p* = 4.9 ⋅ 10^−3^), “neuron projection morphogenesis” (5.4-fold, *p* = 2.3 ⋅ 10^−2^) and “axonogenesis” (6.26-fold, *p* = 1.45 ⋅ 10^−2^). The observed connection between these genes may therefore reflect a role in the differentiation of stem cells into specific types of brain tissue. Hence, it is plausible that perturbations in these differentiating mechanisms result in differentiation of brain cells into cancerous tissue, which would explain why so many of these genes emerge in studies involving gene expression in glioma.

The scope of the method for differential co-expression network analysis presented in this paper is not restricted to only comparing two different tissues within a given organism. In fact, it may be used to compare any two sets of gene expression data for which a comparison might be reasonable: the only criterion is the existence of a viable one-to-one match between the genes in each data set. Possible applications of our method include comparing gene expressions between healthy and sick individuals, comparing samples from experiments with before/after treatments, comparing organisms subjected to different external environments and comparing closely related species with known orthologs.

## Supporting information

S1 Text Example run of the subsampling algorithm. (PDF)

S2 Text Effect of sample size on accuracy of estimates. (PDF)

S3 Text List of software used in our reconstruction and analysis. (PDF)

S4 Text Links to raw data. (PDF)

S5 Text Comparison between the CSD network and a metabolic gene network built from the Human Recon 2 genome-scale reconstruction. (PDF)

S1 Table Top 10 DCe links in the Zhang *et al* [43] regulatory network: C-type, S-type and S-equivalent type. (XLS)

S2 Table An overview of hub nodes for CSD networks generated at different thresholds. (PDF)

S3 Table An overview of KEGG pathways enriched in the CSD network. (XLS)

S4 Table Candidate genes for further glioma association studies. (XLSX)

S1 Fig Distributions of C, S and D scores for our data sets. (TIFF)

S2 Fig Tree diagram of enriched GO biological processes in the C-only network generated using a threshold sample size of 10^5^. (TIFF)

S3 Fig Tree diagram of enriched GO biological processes in the S-only network generated using a threshold sample size of 10^5^. (TIFF)

S4 Fig Tree diagram of enriched GO biological processes in the D-only network generated using a threshold sample size of 10^5^. (TIFF)

S5 Fig Tree diagram of enriched GO biological processes in the combined CSD network generated using a threshold sample size of 10^5^. (TIFF)

S6 Fig Maximum k-cores for various importance value thresholds. (TIFF)
