# Supplementary material for: A composite network of conserved and tissue specific gene interactions reveals possible genetic interactions in glioma
Source: PLoS Comput Biol. 2017 Sep 28;13(9):e1005739. doi: 10.1371/journal.pcbi.1005739 (PMC5634634; doi:10.1371/journal.pcbi.1005739)
Supplement: S1 Text — (PDF) [file pcbi.1005739.s001.pdf]

1. Subsampling

As an aid to understanding the subsampling algorithm, we will show an example run here. Consider a data set consisting of 16 data points (*N = 16*), each labelled by a number. We choose a subsample size *s = 4.*

The first set of subsamples, as specified by the algorithm, becomes as follows:
1, 2, 3, 4; 5, 6, 7, 8; 9, 10, 11, 12; 13, 14, 15, 16;

Drawing subsamples initially containing data point 1 by sequentially adding the lowest numbered data point that hasn't already been subsampled alongside any of the nodes in the subsample, we get the following sample:

1, 5, 9, 13;

Explanation:
1: Starting data point
5: The lowest number not to have already been subsampled with point 1 in previous subsamples. 9: Since 1 has been matched with 2-4 and 5 has been matched with 6-8, the next lowest point not to have already been matched with either 1 or 5 is 9.
13: Similarly, our fourth and final point in the subsample is point 13, as it is the lowest number not to have already been matched with either 1, 5 or 9.

By the same logic, we can draw 3 more subsamples starting from 1.
1, 6, 10, 14; 1, 7, 11, 15; 1, 8, 12, 16

At this point, all data points have already been sampled with point 1, so we cannot draw more samples containing point 1. We therefore proceed to using data point 2 as our starting point, and get the following 4 subsamples:

2, 5, 10, 15; 2, 6, 9, 16; 2, 7, 12, 13; 2, 8, 11, 14

Point 2 having been matched with every other data point, we apply the same process from points 3 and 4, and get 8 more subsamples:

3, 5, 11, 16; 3, 6, 12, 15; 3, 7, 9, 14; 3, 8, 10, 13

4, 5, 12, 14; 4, 6, 11, 13; 4, 7, 10, 16; 4, 8, 9, 15

At this point there are no more valid subsamples to be drawn, as there is no possible combination of 4 data points from which none have previously been subsampled together. In fact, in this case, each data point has been paired exactly once with each of the other data points. This is not an automatic consequence of the algorithm - for different values of *s* and *N,* the algorithm would end when there is no possible choice of *s* data points that have not previously been subsampled together, even if there are individual pairings that have not occurred.
