# Supplementary material for: A composite network of conserved and tissue specific gene interactions reveals possible genetic interactions in glioma
Source: PLoS Comput Biol. 2017 Sep 28;13(9):e1005739. doi: 10.1371/journal.pcbi.1005739 (PMC5634634; doi:10.1371/journal.pcbi.1005739)
Supplement: S2 Text — (PDF) [file pcbi.1005739.s002.pdf]

Effect on sample size on accuracy of estimates

As gene expression is a highly stochastic process, any measurement pertaining to gene expression is sensitive to noise, particularly if evaluated on a data set consisting of few samples. Conversely, these stochastic effects become less significant the larger the sample. For the sake of the following discussion, we use the adjective “fundamental” to describe the hypothetical value a stochastic variable would assume in an infinitely large sample, and for which we seek to provide estimates.

Estimation of correlation:

When using Spearman correlations, it is important to keep in mind that since it only accounts for the relative ranking of data points, there is a significantly higher chance of observing strong correlations when the sample size is small, even if the data is entirely uncorrelated. In an extreme example, two sets of three data points, randomly generated and entirely uncorrelated in theory, have a 1/6th of exhibiting a perfect positive Spearman correlation ($\rho = 1$). More generally, the probability of $\rho = 1$ for two factually uncorrelated series of N points is $1/N!$. It is therefore important that the total sample size is large enough that (a) strong correlations are significant, even after correcting for multiple testing (b) the error in the correlation is minor compared to the domain spanned - that each observed correlation is close to the fundamental value, and consequently, the observed distribution is close to the fundamental distribution.
In order to establish the first criterion, we test:

$$z = \sqrt{\frac{n-3}{1.06} atanh(\rho)}$$ [1]

Which is an approximately normally distributed z-score in the null hypothesis (of uncorrelated sequences). Using an example of a data set containing 50 samples for 10 000 genes (and thus $5 \cdot 10^7$ total gene pairs), a Bonferroni-corrected p-value of 0.05 would require $z > 5.3$, corresponding to $\rho > 0.69$. For a more generous set of 100 samples, the threshold falls to $\rho > 0.53$.
Accurately estimating the error in ρ is less straightforward. However, one approach involves choosing a large data set, and compare the results obtained from smaller subsets of that set, while using the full set as a reference. Specifically, we choose the “Whole blood” data in GTEx v6, as this contains the most data points (338) of any tissue in the GTEx database. We then assess the estimated correlation using 6 different subsets of this data: 3 (non-overlapping) sets

[1] Choi, S. C. (1977). "Tests of Equality of Dependent Correlation Coefficients". *Biometrika*. **64** (3): 645–647. doi:10.1093/biomet/64.3.645.

of 50 points and 3 (non-overlapping) sets of 100 points. We then compare the correlations obtained from the smaller sets with those obtained for a larger 300-point subset, which serves as the reference point - while it may still be subject to noise and not entirely match the root distribution, it should nonetheless allow us to make estimates by extrapolating tendencies over increasing sample sizes. As we intend to investigate the effect of sample size, we decide to settle on a smaller random selection of 100 genes, in order to increase tractability for the larger 300-point data set.

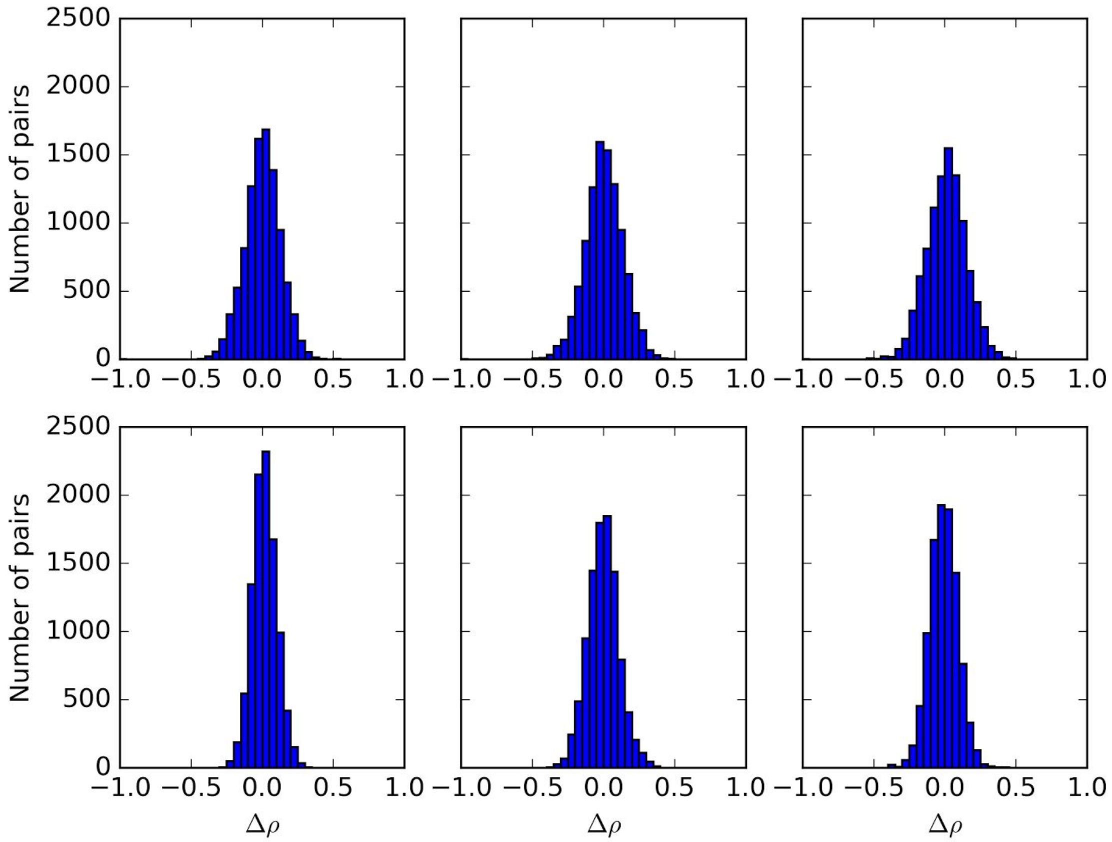

Figure 1: Distribution of error $\Delta\rho$ (relative to 300-point sample) for three 50-point subsets (top) and three 100-point subsets (bottom) in the GTEx v6 “Whole Blood” data set.

Predictably, larger samples are more accurate. We note that even for a sample size of 100, the error in ρ may quite frequently exceed 0.1, which represents at least 10% of any given gene’s correlation value.

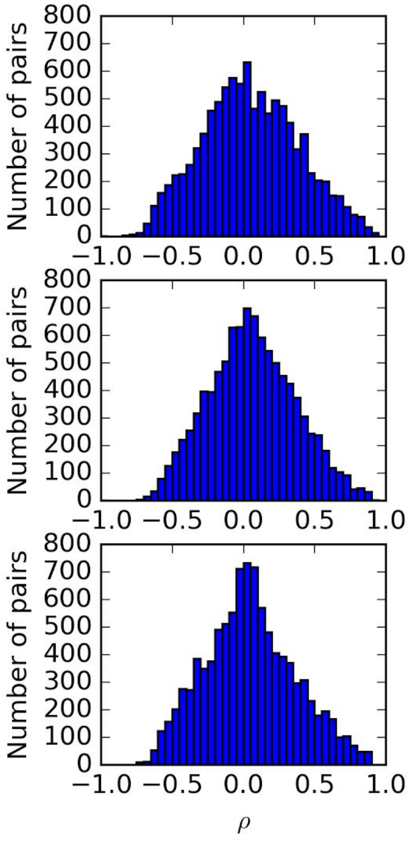

Figure 2: Distribution of correlations for the same three 50-point (top row) and 100-point (middle) data sets as in Figure 1, compared with the correlation for the 300-point data set (bottom).

In this case, it appears from Figure 2 that despite the inherent uncertainty involved in computing correlations from even fairly large samples ($N = 100$), the differences in the resulting distribution are fairly minimal, with similar variation between samples of different sizes as between those of the same size.

Estimation of variance:

The relationship between the accuracy of the variance estimate and the total sample size is somewhat simpler. Since all subsamples consist of data points drawn from (a subset of) the same fundamental distribution, the standard error of the correlation for a given subsample depends only the size of the subsample (provided the whole sample approximates the fundamental distribution). In turn, this means that for a set of subsamples, the variance of correlations depends only on the subsample size, while being independent of the total sample size. Since the subsample size is manually specified and not fixed relative to the total sample size, we can easily ensure that we use the same subsample size for both conditions, and consequently avoid an undue bias towards the variance exhibited under one specific condition.

However, as the total number of subsamples that can be generated strongly depends on the number of total samples, it is important that we have enough subsamples to ensure that the estimated variance is accurate.

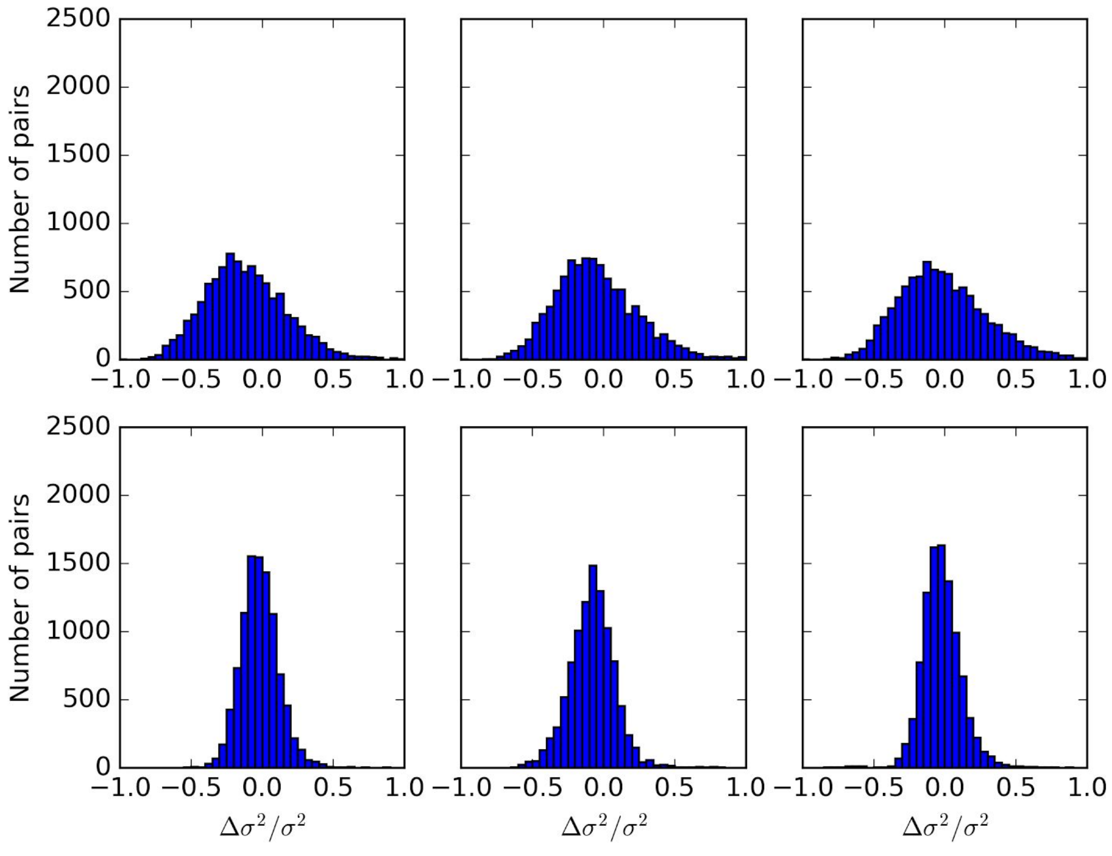

Figure 3: Distribution of relative error $\Delta\sigma^2/\sigma^2$ (relative to 300-point sample) for the three 50-point subsets (top) and three 100-point subsets (bottom) in the GTEx v6 “Whole Blood” data set.

Figure 3:

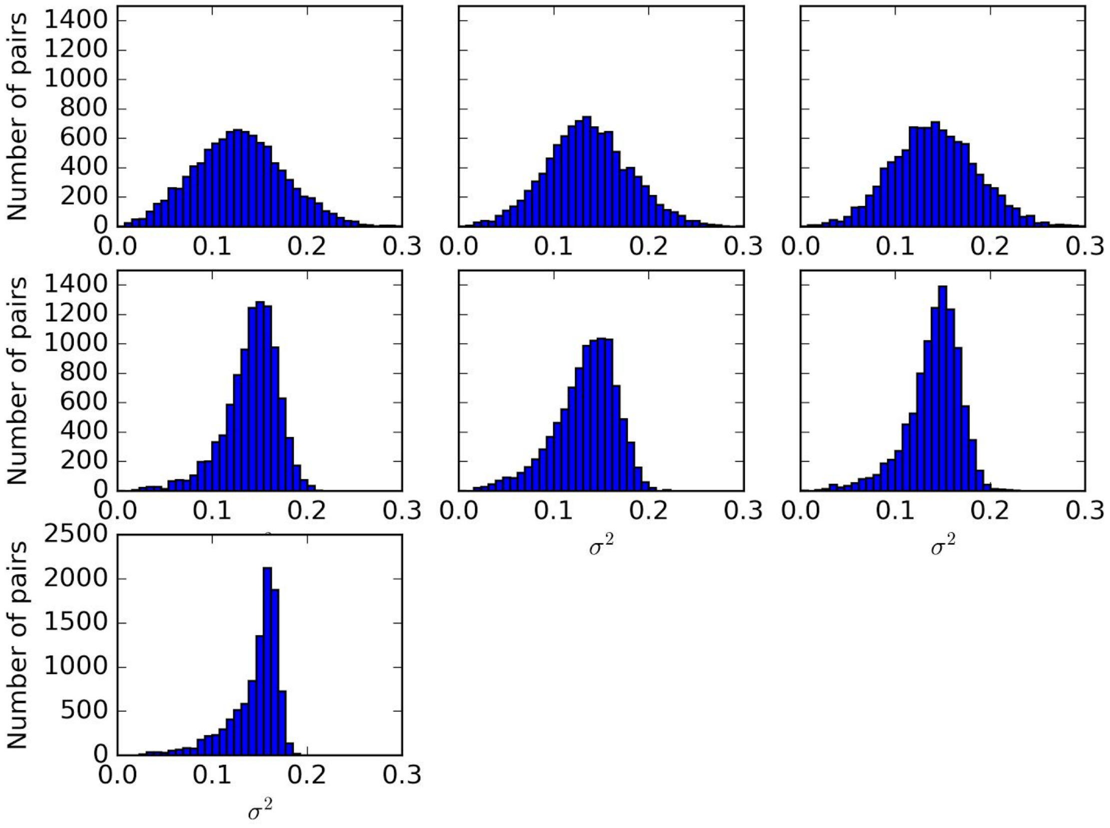

Figure 4: Distribution of correlations for the same three 50-point (top row) and 100-point (middle) data sets as in Figures 1, 2, and 3, compared with the correlation for the 300-point data set (bottom).

As we see in Figures 3 and 4, σ is quite susceptible to changes in the sample size, more so than ρ, made all the more apparent by the fact that the root distribution appears to transition from a symmetric bell shape to a negative skew as the sample size increases. The mean variance increases faintly with N; for $N = 50$, we find $\overline{\sigma^2} = 0.128$, $\overline{\sigma^2} = 0.136$ and $\overline{\sigma^2} = 0.141$; for $N = 100$, $\overline{\sigma^2} = 0.142$, $\overline{\sigma^2} = 0.133$, $\overline{\sigma^2} = 0.140$; for $N = 300$, $\overline{\sigma^2} = 0.145$. This effect, however, is not statistically significant ($p = 0.174$, Spearman test).
