# Supplementary material for: A composite network of conserved and tissue specific gene interactions reveals possible genetic interactions in glioma
Source: PLoS Comput Biol. 2017 Sep 28;13(9):e1005739. doi: 10.1371/journal.pcbi.1005739 (PMC5634634; doi:10.1371/journal.pcbi.1005739)
Supplement: S3 Text — (PDF) [file pcbi.1005739.s003.pdf]

CSD software

Source code used in the generation of CSD networks from expression data can be found on https://github.com/andre-voigt/CSD

Third-party software resources:

Synthetic expression data: GeneNetWeaver (http://gnw.sourceforge.net/)

Network visualization: Cytoscape (http://cytoscape.org/)

Network analysis: NetworkX (https://networkx.github.io/)

GO functional enrichment: GOrilla (http://cbl-gorilla.cs.technion.ac.il/)

KEGG pathway enrichment: Enrichr (http://amp.pharm.mssm.edu/Enrichr/)

OMIM disease enrichment: Enrichr (http://amp.pharm.mssm.edu/Enrichr/)
