# Supplementary material for: A composite network of conserved and tissue specific gene interactions reveals possible genetic interactions in glioma
Source: PLoS Comput Biol. 2017 Sep 28;13(9):e1005739. doi: 10.1371/journal.pcbi.1005739 (PMC5634634; doi:10.1371/journal.pcbi.1005739)
Supplement: S4 Text — (PDF) [file pcbi.1005739.s004.pdf]

2. CSD network information

The following data for the CSD network is available online at https://www.ntnu.edu/almaaslab:

- List of genes present in the CSD network
- Complete list of genes with gene-expression data
- Edge list for the CSD network, with interaction type and score, as well as correlation and variance for each of the tissues.
- Full set of correlations and variances, as well as C, S and D scores, for all background genes.
