# Supplementary material for: A composite network of conserved and tissue specific gene interactions reveals possible genetic interactions in glioma
Source: PLoS Comput Biol. 2017 Sep 28;13(9):e1005739. doi: 10.1371/journal.pcbi.1005739 (PMC5634634; doi:10.1371/journal.pcbi.1005739)
Supplement: S5 Text — (PDF) [file pcbi.1005739.s005.pdf]

3. Metabolic networks

Method

In order to determine if the observed CSD-links reflect metabolic relationships between genes, we generated a metabolic gene network from the Recon 2 model of human metabolism. The metabolic gene network was created as part of a multi-layer network, with two additional auxiliary networks:

- First, we create a reaction-centric network (RCN). Nodes represent reactions, and connect to another reaction if they both involve at least one common metabolite. In order to filter out connections from ubiquitous metabolites such as ATP or water, we require the shared metabolite to be used or produced by less than 10 different reactions.
- We then create a bipartite gene/reaction-centric network. Nodes may represent either genes or metabolites. Links connect genes to those reactions that rely on their gene product to run. There are no connections between pairs of genes or pairs of reactions - each link connects a reaction to a gene.
- Finally, we create the metabolic gene network (MGN). Nodes correspond to genes, and connect to each other if they share at least one nearest neighbor in the bipartite network (step 2), or if any of their nearest neighbors in the bipartite network are nearest neighbors in the reaction-centric network (step 1).

In short, the MGN connects genes if they are necessary for the production and/or consumption of at least one common (non-ubiquitous) metabolite.

Results

The MGN consists of 1117 genes, (coincidentally) connected by exactly 5000 edges.

We do not find any significant overlap (beyond that expected by random chance) between the MGN and the CSD with respect to number of nodes or links. Of the 1675 genes present in the Recon 2 model, 1629 are present in the original GTEx data sets (18453 genes). 1086 of these genes are also nodes in the MGN.

Of these 1086 genes, 110 are also contained in the CSD-network (which contains a total of 1798 genes). In other terms, the MGN contains 5.89% of genes in the base expression data and 6.11% of the genes in the CSD-network. While metabolically linked genes are slightly overrepresented in the CSD-network, $p \approx 0.31$ (hypergeometric test), and so this enrichment cannot be said to be statistically significant.

Of the 2351 links in the CSD-network, 12 connect genes which are both found in the MGN. This is not significantly higher than the expected number (8, $p \approx 0.2$) of links between 110 randomly marked genes in the underlying network. Of the 12 gene pairs in the MGN corresponding to CSD-links, 4 have connecting paths (the remaining 8 pairs consist of nodes from separate connected components). These 4 pairs (path lengths in parentheses) are AKR1B1 and DDC (3), B3GALT5 and MGAT4A (4), MGAT4A and PDE7B (9), MT-CO1 and MT-CO2 (1).

In summary, we do not find any substantial overlap between the CSD-network and the MGN.
