# Supplementary material for: A composite network of conserved and tissue specific gene interactions reveals possible genetic interactions in glioma
Source: PLoS Comput Biol. 2017 Sep 28;13(9):e1005739. doi: 10.1371/journal.pcbi.1005739 (PMC5634634; doi:10.1371/journal.pcbi.1005739)
Supplement: S2 Table — (PDF) [file pcbi.1005739.s007.pdf]

Conservation of prominent nodes

\Cutoff<br>Rank\	1e -4	8e-5	5e-5	2e-5	1e-5	8e-6	5e-6	2e-6	1e-6
1	UBQLN1 (84)	UBQLN1 (75)	ATP6V1 C1 (53)	UBQLN1 (31)	UBQLN1 (22)	UBQLN1 (16)	TRIM37 (11)	FA2H (6)	RAB39 B (4)
2	ATP6V1 C1 (82)	ATP6V1 C1 (72)	UBQLN1 (51)	ATP6V1 C1 (29)	TRIM37 (17)	TRIM37 (15)	UBQLN1 (11)	TRIM3 7 (6)	C1QB (3)
3	NBEA (64)	NBEA (51)	LMBR1 (38)	TRIM37 (21)	FAXC (15)	FAXC (12)	FAXC (10)	PPP3C B (6)	TRIM37 (3)
4	LMBR1 (56)	CAB39 (50)	FAXC (36)	PPP3CB (19)	PPP3CB (14)	PPP3CB (11)	PPP3CB (9)	UBQLN 1 (6)	FAXC (3)
5	MAP7D2 (56)	LMBR1 (49)	NBEA (36)	SCN2A (19)	ATP6V1 C1 (14)	ATP6V1 C1 (11)	ATP6V1 C1 (8)	MYRF (5)	DLGAP 4 (2)
6	TRIM37 (55)	PPP3CB (48)	TRIM37 (35)	FAXC (18)	FAM8A1 (12)	ATL1 (10)	RAB39B (8)	FAXC (5)	MYRF (2)
7	CAB39 (55)	MAP7D2 (48)	MAP7D2 (34)	NBEA (17)	ATL1 (11)	MAP7D2 (10)	SCN2A (7)	PCMT1 (4)	SNAP2 5 (2)
8	FAXC (55)	FAXC (46)	GUCY1B 3 (33)	ITFG1 (17)	NBEA (11)	RTN3 (9)	MYRF (7)	RAB39 B (4)	FA2H (2)
9	PPP3CB (52)	TRIM37 (45)	PPP3CB (31)	FAM8A1 (16)	MAP7D2 (11)	NBEA (9)	ATL1 (7)	MAPK9 (4)	FAM13 4A (2)
10	SCN2A (52)	UBE2K (45)	UBE2K (29)	MAP7D2 (16)	UBE2K (10)	FAM8A1 (9)	MAP7D2 (7)	CAB39 (4)	AP2M1 (2)

S2 Table a): Top 10 nodes by degree (specified in parentheses) in C-networks generated with 9 different cut-offs.

\Cutoff Rank\	1.00E+04	8.00E+05	5.00E+05	2.00E+05	1.00E+05	8.00E+06	5.00E+06	2.00E+06	1.00E+06
1	GPR101 (289)	GPR101 (248)	GPR101 (172)	POU3F4 (81)	POU3F4 (44)	POU3F4 (37)	POU3F4 (29)	ECE2 (13)	ECE2 (7)
2	POU3F4 (245)	POU3F4 (210)	POU3F4 (146)	GPR101 (76)	GPR101 (44)	GPR101 (33)	ECE2 (23)	GPR101 (12)	GPR101 (6)
3	ECE2 (192)	ECE2 (170)	ECE2 (110)	ECE2 (56)	ECE2 (33)	ECE2 (29)	GPR101 (22)	DIRAS3 (10)	C6orf141 (5)
4	DESI2 (157)	DESI2 (144)	DESI2 (104)	DESI2 (51)	DESI2 (30)	DIRAS3 (26)	DIRAS3 (20)	DESI2 (9)	SNCAIP (4)
5	TLE4 (150)	TLE4 (123)	DIRAS3 (85)	DIRAS3 (50)	DIRAS3 (27)	DESI2 (24)	DESI2 (18)	C6orf141 (8)	DESI2 (4)
6	DIRAS3 (135)	DIRAS3 (112)	USP47 (76)	RASL11 A (39)	RASL11A (22)	RASL11 A (19)	RASL11 A (14)	POU3F4 (8)	LPL (3)
7	USP47 (128)	USP47 (108)	RASL11 A (75)	USP47 (36)	LPL (20)	LPL (17)	C6orf141 (12)	PDE7B (7)	DIRAS3 (3)
8	LAMC2 (119)	LAMC2 (96)	TLE4 (75)	TLE4 (31)	RPS6KA 5 (17)	TLE4 (15)	LPL (11)	LPL (6)	HNRNPC (3)
9	RASL11 A (109)	PELI1 (94)	SLC30A3 (67)	LAMC2 (27)	TLE4 (17)	C6orf141 (14)	TLE4 (10)	LAMC2 (5)	PKDCC (2)
10	SLC30A3 (108)	RASL11 A (94)	LAMC2 (61)	LPL (27)	C6orf141 (17)	SNCAIP (14)	SLC30A3 (9)	SNCAIP (5)	SLC30A3 (2)

S2 Table b): Top 10 nodes by degree (specified in parentheses) in S-networks generated with 9 different cut-offs.

\Cutoff Rank\	1.00E+04	8.00E+05	5.00E+05	2.00E+05	1.00E+05	8.00E+06	5.00E+06	2.00E+06	1.00E+06
1	FOXO1 (869)	FOXO1 (770)	FOXO1 (588)	FOXO1 (353)	FOXO1 (235)	FOXO1 (207)	FOXO1 (144)	FOXO1 (83)	FOXO1 (46)
2	CARHSP1 (695)	CARHSP1 (605)	CARHSP1 (451)	CARHSP1 (222)	ATP11C (125)	ATP11C (99)	CARHSP1 (57)	CARHSP1 (24)	CARHSP1 (14)
3	ATP11C (592)	ATP11C (532)	ATP11C (413)	ATP11C (216)	CARHSP1 (117)	CARHSP1 (94)	ATP11C (56)	ATP11C (23)	ATP11C (9)
4	PBX3 (446)	PBX3 (373)	PBX3 (237)	PBX3 (95)	PBX3 (37)	PBX3 (24)	PBX3 (15)	EYA1 (4)	EYA1 (3)
5	DDO (384)	DDO (324)	DDO (194)	DDO (69)	DDO (19)	DDO (16)	DDO (14)	LPL (3)	LPL (2)
6	GSTM2 (289)	GSTM2 (243)	GSTM2 (144)	GSTM2 (44)	EYA1 (18)	LPL (15)	EYA1 (12)	AP2M1 (3)	ST6GALNAC5 (2)
7	LHFPL3 (217)	LHFPL3 (188)	LHFPL3 (103)	EYA1 (35)	LPL (17)	EYA1 (14)	GSTM2 (7)	TMEM255A (2)	IL1RL2 (1)
8	EYA1 (194)	EYA1 (161)	EYA1 (97)	LHFPL3 (34)	GSTM2 (14)	TMEM255A (11)	LPL (6)	HUNK (2)	RIBC1 (1)
9	TPD52L1 (156)	LPL (125)	LPL (84)	LPL (31)	LHFPL3 (13)	GSTM2 (9)	LHFPL3 (5)	H2AFJ (2)	ZFYVE9 (1)
10	LPL (151)	TPD52L1 (119)	TPD52L1 (67)	TMEM255A (21)	TMEM255A (12)	PPP1R1B (8)	DUSP3 (5)	C10orf35 (2)	TPI1 (1)

S2 Table c): Top 10 nodes by degree (specified in parentheses) in D-networks generated with 9 different cut-offs.
