# Supplementary figures and images for: A composite network of conserved and tissue specific gene interactions reveals possible genetic interactions in glioma
Source: PLoS Comput Biol. 2017 Sep 28;13(9):e1005739. doi: 10.1371/journal.pcbi.1005739 (PMC5634634; doi:10.1371/journal.pcbi.1005739)

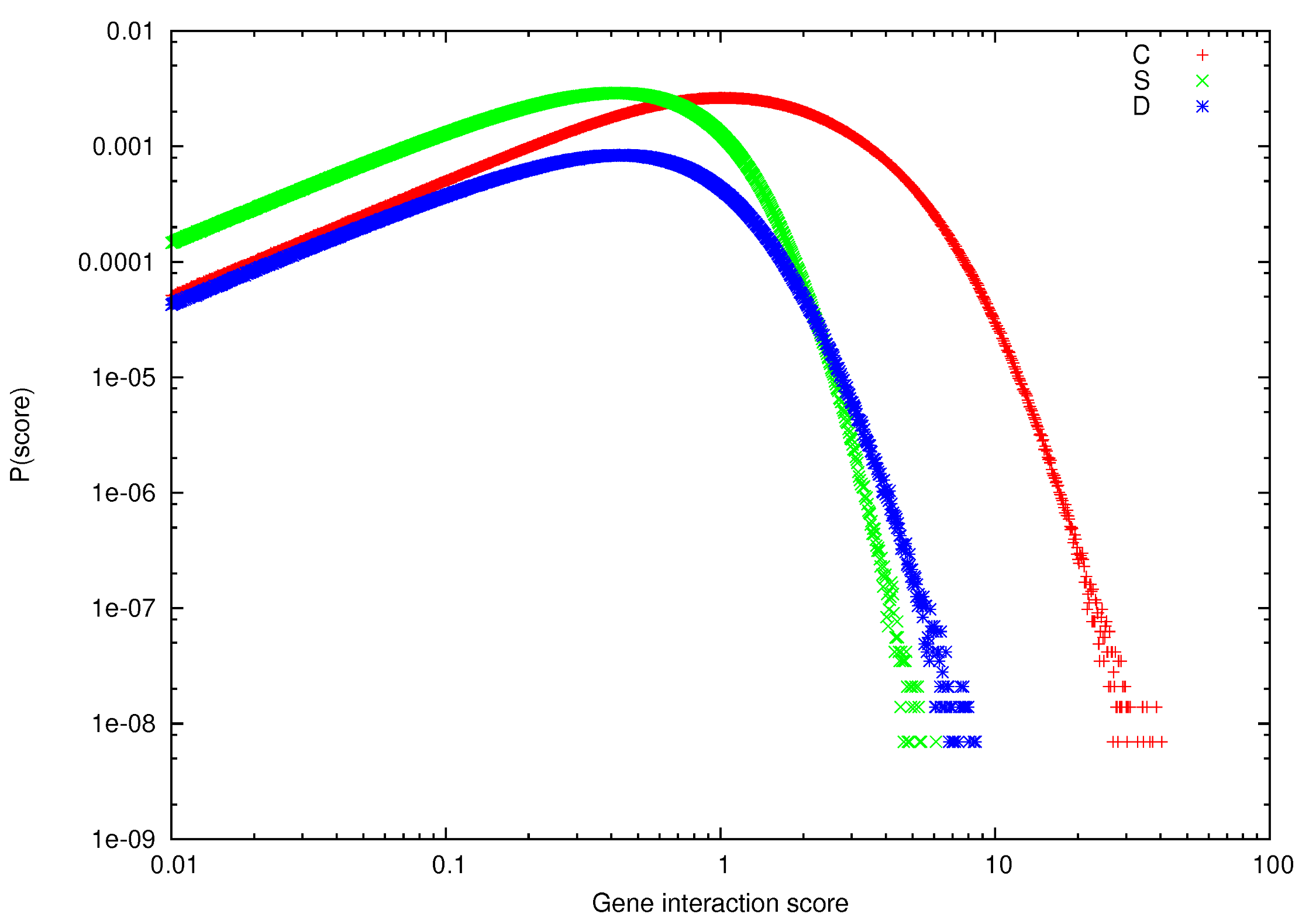

Supplement: S1 Fig — (TIFF) [file pcbi.1005739.s010.tiff]

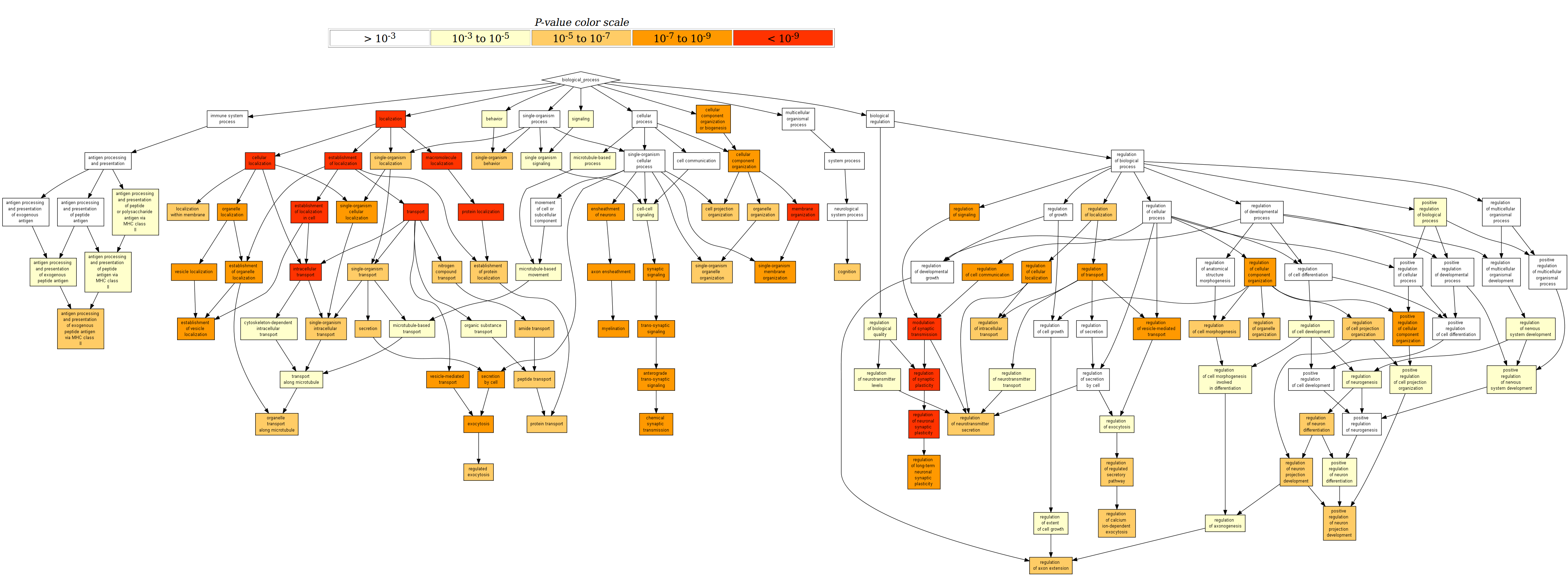

Supplement: S2 Fig — (TIFF) [file pcbi.1005739.s011.tiff]

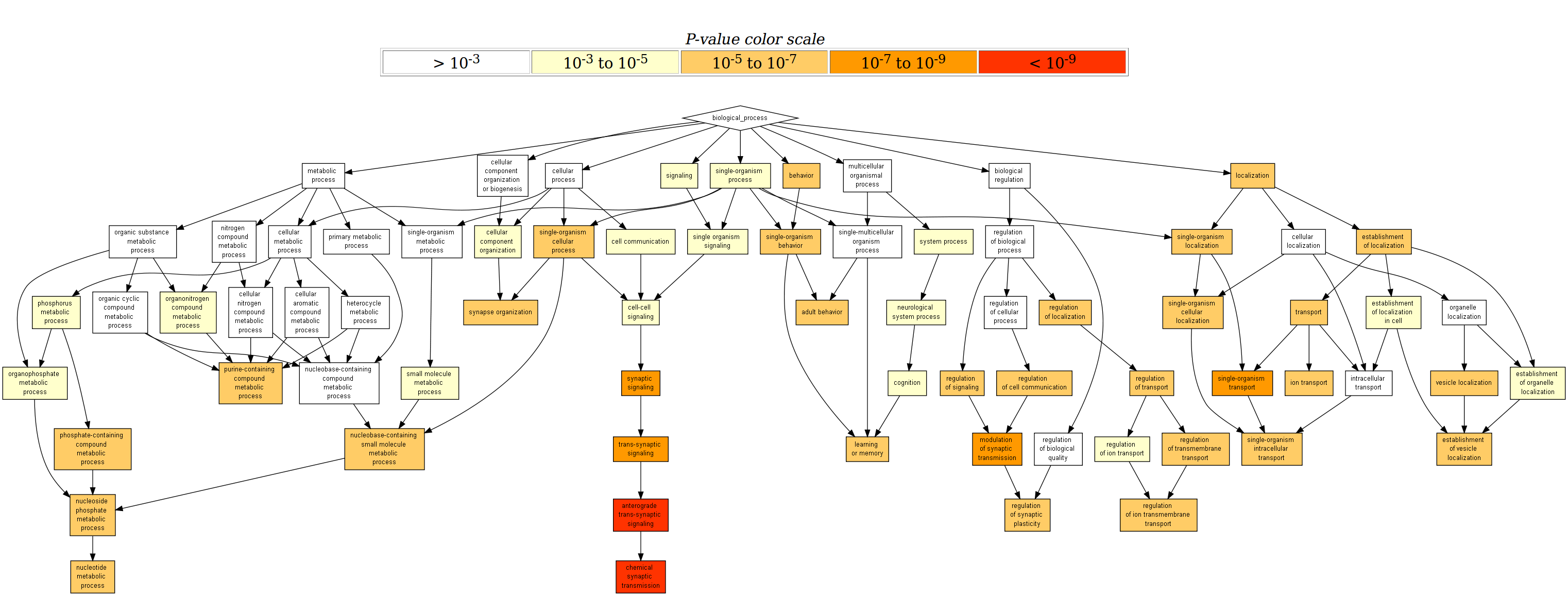

Supplement: S3 Fig — (TIFF) [file pcbi.1005739.s012.tiff]

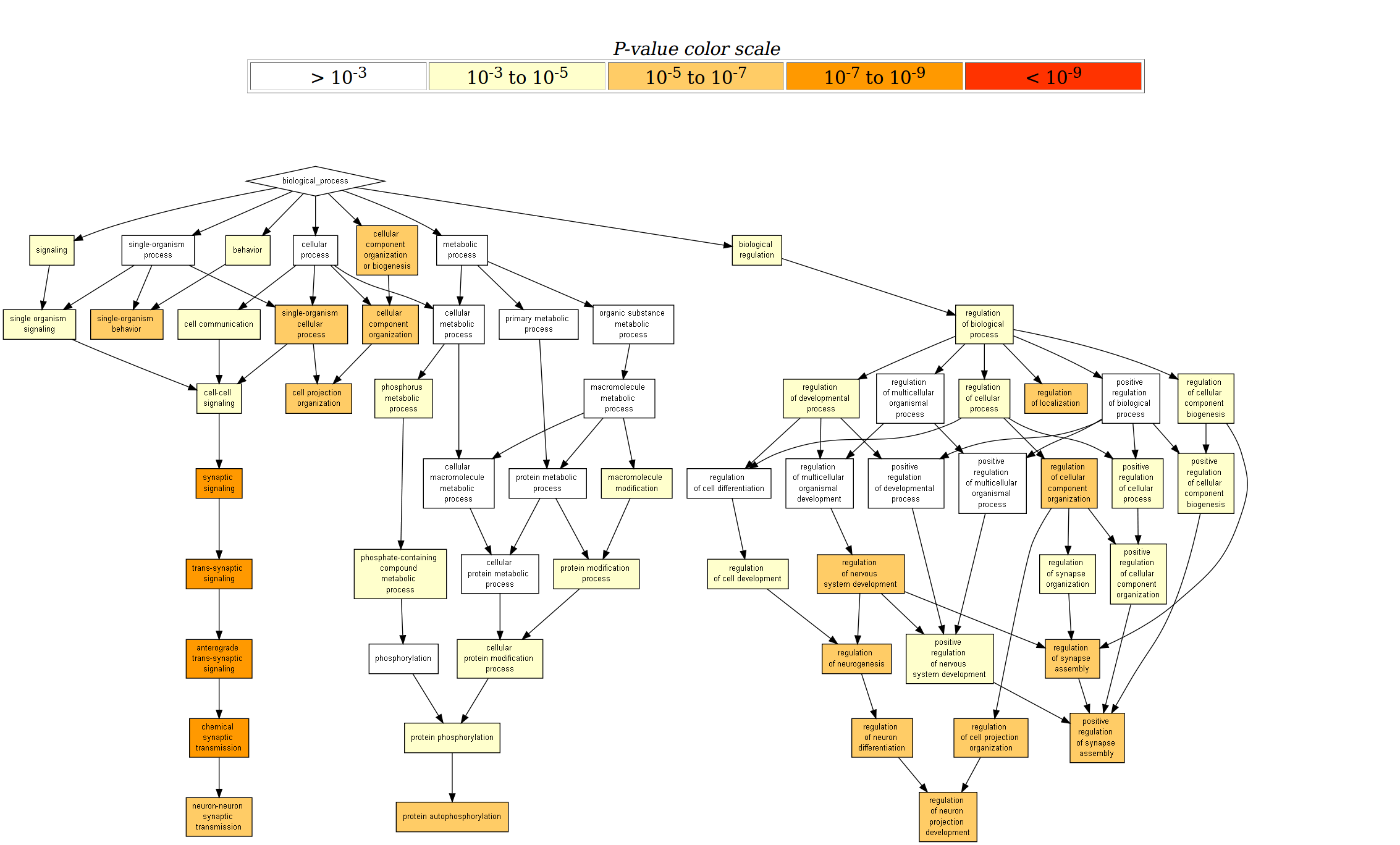

Supplement: S4 Fig — (TIFF) [file pcbi.1005739.s013.tiff]

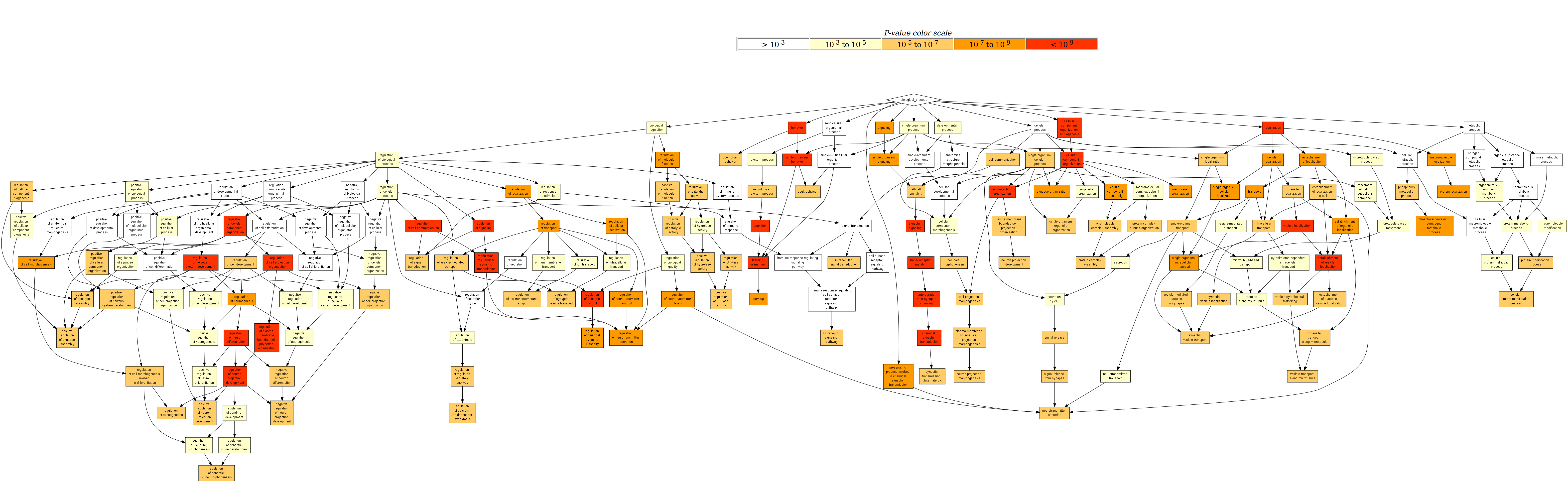

Supplement: S5 Fig — (TIFF) [file pcbi.1005739.s014.tiff]

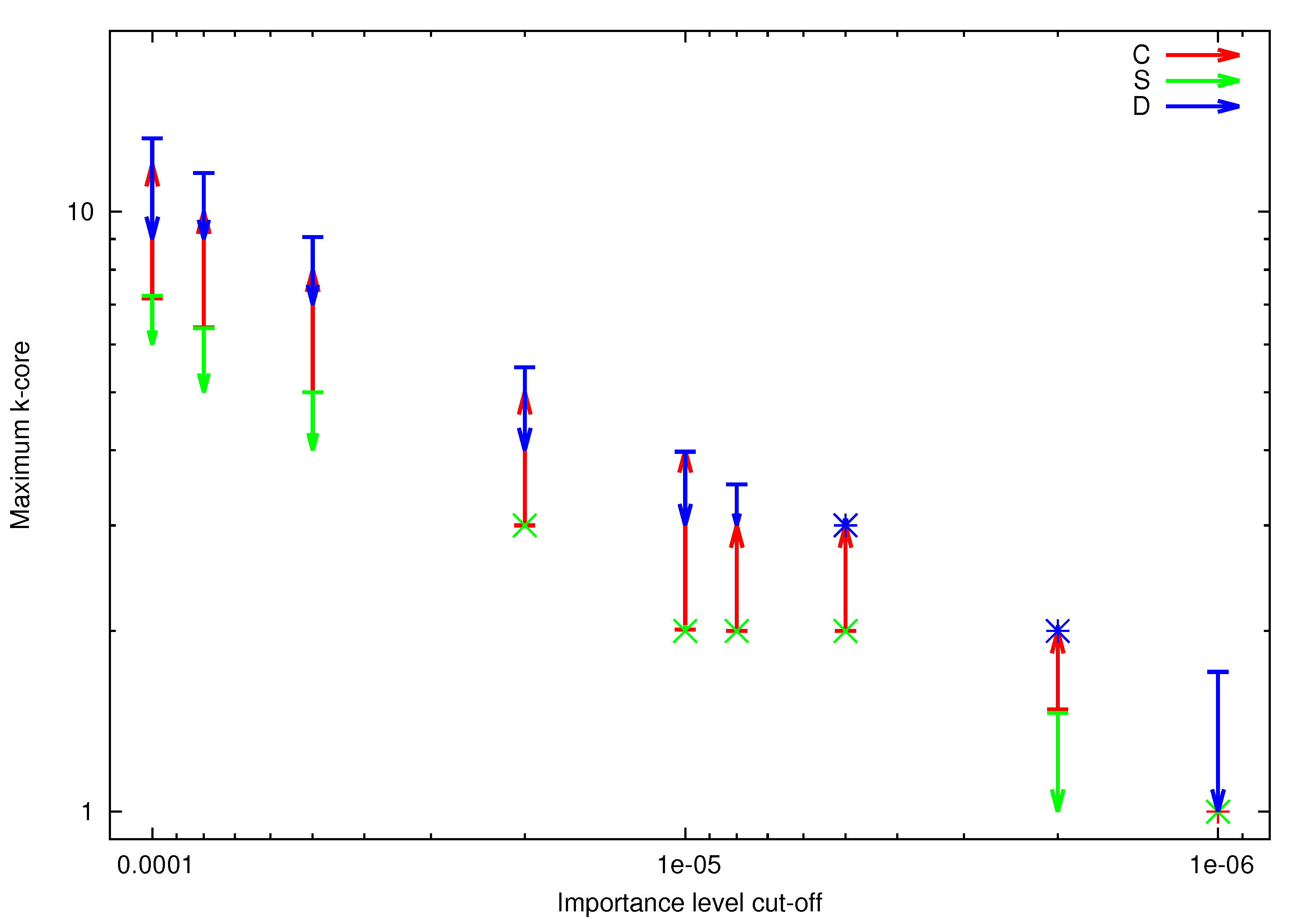

Supplement: S6 Fig — (TIFF) [file pcbi.1005739.s015.tiff]
